# Defining immune correlates during latent and active chlamydial infection in sheep

**DOI:** 10.1186/s13567-020-00798-6

**Published:** 2020-06-01

**Authors:** Sean R. Wattegedera, Morag Livingstone, Stephen Maley, Mara Rocchi, Susan Lee, Yvonne Pang, Nick M. Wheelhouse, Kevin Aitchison, Javier Palarea-Albaladejo, David Buxton, David Longbottom, Gary Entrican

**Affiliations:** 1 Moredun Research Institute, International Research Centre, Pentlands Science Park, Bush Loan, Penicuik, Scotland EH26 0PZ UK; 2 The Roslin Institute and Royal (Dick) School of Veterinary Studies, The University of Edinburgh, Easter Bush, Midlothian, Scotland EH25 9RG UK; 3 School of Applied Sciences, Napier University, Edinburgh, Scotland EH11 4BN UK; 4 Biomathematics and Statistics Scotland, JCMB, The King’s Buildings, Peter Guthrie Tait Road, Edinburgh, Scotland EH9 3FD UK

## Abstract

Ovine enzootic abortion (OEA) caused by the obligate intracellular bacterial pathogen *Chlamydia abortus* (*C. abortus*), is an endemic disease in most sheep-rearing countries worldwide. Following infection, *C. abortus* establishes a complex host–pathogen interaction with a latent phase in non-pregnant sheep followed by an active disease phase in the placenta during pregnancy leading to OEA. Improved knowledge of the host–pathogen interactions at these different phases of disease will accelerate the development of new diagnostic tests and vaccines to control OEA. Current evidence indicates that cellular immunity is essential for controlling *C. abortus* infection. We have previously described a model of mucosal (intranasal) infection of non-pregnant sheep with *C. abortus* that replicates the latent and active phases of OEA. We have investigated antigen-specific recall responses of peripheral blood mononuclear cells (PBMC) in sheep infected with *C. abortus* via the intranasal route to determine how these change during the latent and active phases of disease. By analysing cytokines associated with the major CD4^+ve^ T_helper_ (T_h_) cell subsets (Interferon-gamma (IFN-γ)/T_h_1; Interleukin (IL)-4/T_h_2; IL-17A/T_h_17; IL-10/T_regulatory_), we show that there is selective activation of PBMC producing IFN-γ and/or IL-10 during the latent phase following infection. These cytokines are also elevated during the active disease phase and while they are produced by sheep that are protected from OEA, they are also produced by sheep that abort, highlighting the difficulties in finding specific cellular immunological correlates of protection for complex intracellular pathogens.

## Introduction

*Chlamydia abortus* (*C. abortus*) is a common infectious cause of ovine abortion world-wide (with the exception of Australia and New Zealand) despite the availability of commercial vaccines [1, 2]. The disease is variously referred to as ovine enzootic abortion (OEA), enzootic abortion of ewes (EAE), ovine chlamydiosis and chlamydial abortion. A number of safety concerns, vaccination failure and transmission of disease to naïve animals have all been linked to the current commercial live-attenuated vaccines [3–6]. The lack of a serological DIVA (Differentiating Infected from Vaccinated Animals) test is a further complicating factor in the implementation of effective disease control strategies [7]. The development of new control strategies incorporating a DIVA approach is hampered by a relatively poor understanding of how immune responses at different stages of infection relate to disease outcome.

Sheep become infected with *C. abortus* via the oronasal route by contact with pasture contaminated with abortive placental membranes and fluids, aborted and pre-term foetuses or infected lambs that survive [1]. Primary infection of non-pregnant sheep can result in an asymptomatic, latent infection that leads to colonisation of placental trophoblast cells in the subsequent pregnancy, destruction of the chorionic epithelium and abortions that manifest in the last 2–3 weeks of gestation [1, 8]. These events appear to be synchronised with the later stages of pregnancy as placental lesions have only been reliably observed from day 90 gestation onwards [9, 10].

The host immune responses during these different stages of infection and disease are not well understood. To investigate this we have experimentally reproduced a model of latent *C. abortus* infection in non-pregnant sheep which results in abortion in the subsequent pregnancy [11]. We have so far failed to identify a correlation between the humoral immune response, antigen-specific interferon-gamma (IFN-γ) production by peripheral blood mononuclear cells (PBMC) and disease outcome. Here, we expand the breadth and depth of our immunological analyses of the cellular immune response following intranasal infection to include antigen-specific proliferation of PBMC and the concomitant expression of key cytokines that are associated with activation of the major CD4^+ve^ T_helper_ (T_h_) cell subsets (IFN-γ, interleukin (IL)-4, IL-10 and IL-17A) to gain a greater understanding of latency and provide insight into protective immune mechanisms that could be exploited for the design and development of the next generation of safer, subunit vaccines to OEA.

## Materials and methods

**Figure 1:**
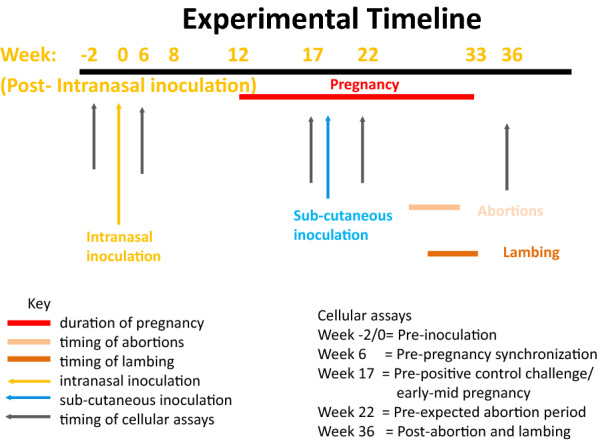
**Experimental overview of cellular assays in relation to experimental inoculations with*Chlamydia abortus*and subsequent pregnancy.** The experimental timeline of 36 weeks represented in the diagram with the timing of the cellular assays carefully selected to monitor responses pre-inoculation to post-parturition. Five time points were chosen for this, to correspond to pre-inoculation (week 0), pre-synchronisation and mating (week 6), pre-positive sub-cutaneous (s/c) control (group 5) challenge to coincide with early pregnancy of the intranasal inoculation (i/n) groups (week 17), pre-expected abortion period (week 22) and finally post-abortion/lambing (week 36) where these are represented by grey arrows. The week of the study is anchored by the i/n at week 0. For clarity, the pre-inoculation bleed is referred to as occurring at week 0 and not week −2, consistent with the paper describing the experimental model [8]. The five cellular bleeds for the s/c positive control Group 5 are simultaneously referred to the following week numbers: −21, −11, 0, 5 and 19. The orange arrow represents the timing of i/n with *Chlamydia abortus* (*C. abortus*) of the sheep: Group 1, 5 × 10^3^ infection forming units (IFU)/mL, low dose; Group 2, 5 × 10^5^ IFU/mL, medium dose; Group 3, 5 × 10^7^ IFU/mL, high dose group and group 4, sham inoculation group (no *C. abortus*). The blue arrow represents the subcutaneous inoculation at day 70 of gestation of group 5 with 2 × 10^6^ IFU/mL over the left pre-femoral lymph node. Red line represents the duration of pregnancy from week 12 to week 33. The orange line represents when the abortions took place and the brown line the period of time when the lambing occurred in all groups.

The experimental animal methodology has been described previously to include all clinical, histopathological and microbiological data along with some limited immunological analyses. Note that all data were presented by experimental inoculation group only, without reference to disease outcome [11]. An overview of the experimental design and timeline of sample points for the cellular immunology assays is shown in Figure 1.

### Animal study ethical approval

This study was approved by the Moredun Animal Welfare Ethical Review Body (permit number: E37/07) and was compliant with the UK Animal (Scientific Procedures) Act 1986. All animals were monitored throughout the study for any clinical signs at least three times daily and all findings recorded. Any animal found to be suffering or requiring treatment was given appropriate veterinary care in accordance with standard veterinary practice.

### Selection and assignment of experimental animals

As we have previously shown that there is considerable variation in both mitogen-driven and antigen-specific cellular responses in outbred sheep [12, 13], animals were pre-screened for baseline immune responses and then allocated to groups to avoid the introduction of bias between groups. On that basis, seventy-nine ewes were selected from a cohort of ninety-four Scottish Blackface sheep (3 to 5 years of age) seronegative for *C. abortus* (rOMP90 ELISA, [14]) by evaluation of cellular responses to *C. abortus* antigen and to the mitogen Concanavalin A (ConA; ICN Biochemicals, Cleveland, OH, USA) 2 weeks prior to challenge. As not all animals become pregnant, it was imperative to start with more sheep than required per group to ensure statistically robust numbers for the duration of the experiment.

Briefly, PBMC were subject to in vitro lymphocyte stimulation assays using UV-killed *C. abortus* antigen, mock antigen (HEp-2 cell lysate), ConA as a positive control and culture medium as a negative control. Culture supernatants were analysed and quantified for the presence of IFN-γ by ELISA [13] and cellular proliferation was measured in duplicate cultures by ^3^H-Thymidine incorporation. Sheep were excluded on the basis of defined cut-off values to the recall chlamydial antigen and mitogen ConA, as outlined in Table 1. The animals were ranked according to responsiveness to ConA (IFN-γ production and cellular proliferation) and then randomly allocated across the five groups. Subsequently, twelve of these sheep did not become pregnant and were removed from the study, leaving a final cohort of sixty-seven.

**Table 1:** **Cellular response cut-off values for animal selection**

Treatment	IFN-γ production cut-off value (pg/mL)	Cellular proliferation cut-off value (counts per minute)
Medium	Above 250	Above 30 000
HEp-2	Above 250	Above 30 000
UV-*Chlamydia abortus*	Above 1100	Above 70 000
Concanavalin A	Below 450	Below 150 000

For each lymphocyte stimulation assay in vitro treatment, defined cut-offs for animal selection were defined for IFN-γ production (measured on culture supernatant samples collected at 96 h as described in section “Cytokine ELISAs”) and for cellular proliferation (measured for the last 18 h of the 120 h culture as described in section “Cell proliferation assays”). For medium, HEp-2 and UV-*Chlamydia abortus* antigen the cut-off values for exclusion of outliers were above the values indicated in the IFN-γ production and cellular proliferation. For Concanavalin A, the cut-off values for exclusion of outliers were below the values indicated for IFN-γ and cellular proliferation, respectively.

### Intranasal inoculation (Nasal-associated Lymphoid Tissue challenge) of non-pregnant sheep with *Chlamydia abortus* and sub-cutaneous inoculation of pregnant sheep with *C. abortus*

Non-pregnant sheep were inoculated intranasally (i/n) over the pharyngeal mucous membrane using an adapted disposable syringe attached to a plastic tube with a perforated end cap to ensure even coating of the pharyngeal cavity, as previously described [11] with one of three doses of yolk-sac grown *C. abortus*: 5 × 10^3^ (low dose; *n* = 16), 5 × 10^5^ (medium dose; *n* = 18) and 5 × 10^7^ (high dose; *n* = 19) inclusion forming units (IFU) (groups 1–3, 21 animals each); an additional eight animals received an intranasal inoculation of yolk sac (sham control; group 4). Six sheep assigned to the positive control group were left untreated at this stage (group 5). All sheep were synchronised for oestrus following application of progesterone sponges (Veramix, Upjohn Ltd, Crawley, UK) and then batch mated with rams, as previously described [11]. At 70 days of gestation (dg) the group 5 sheep were inoculated subcutaneously (s/c; group 5 will also be referred to as s/c control group) as previously described [11]. All animals were housed in separated groups to avoid cross-contamination. Rectal temperatures were recorded daily in groups 1–4 on the day of i/n and for a total of 27 consecutive days. Abortions and lamb viability were monitored and samples were collected for pathological, microbiological and immunological examination, as described previously [11].

### Preparation of UV-inactivated *Chlamydia abortus* antigen

The antigen used in the recall assays was UV-inactivated *C. abortus* strain S26/3 elementary body (EB) and reticulate body (RB) mixture [15]. The strain was propagated in HEp-2 cells using Iscove’s Modified Dulbecco’s Medium (IMDM) supplemented with 10% heat-inactivated (ΔH) fetal bovine serum (FBS, PAA Gold USA Origin, PAA Hanninger, Austria) and 2 mM l-glutamine. Once confluent, cells were infected with *C. abortus* strain S26/3 inoculum for 4 h at 37 °C under 5% CO_2._ The inoculum was then removed, replaced with IMDM 2% ΔH FBS and cultured for 72 h in a humidified incubator at 37 °C under 5% CO_2_. The asynchronous *Chlamydia* culture was harvested by mechanical disruption using sterile glass beads. The supernatant was clarified of cellular debris by centrifugation at 900 × *g* for 10 min at 4 °C and transferred to small microfuge tubes for a final centrifugation step at 10 000 × *g* for 10 min at 4 °C. The pelleted chlamydial EBs were resuspended in sucrose-phosphate-glutamate buffer (SPG) [16] and stored at -70 °C. The chlamydial antigen was UV-inactivated with 2000 rads using a Uvitec Cross-Linker CL-E508.G 9 (Thermofisher, Cambridge, UK) and titrated in cellular assays with PBMC and assessed for an optimal IFN-γ concentration in culture supernatants, as described previously [11].

### Blood collection and peripheral blood mononuclear cell (PBMC) preparation

Ten millilitres of venous blood was collected into vacutainers on multiple occasions for the preparation of serum (Becton–Dickinson, Cambridge, UK) for the detection of humoral responses; twenty millilitres of additional blood were also collected into vacutainers containing lithium heparin (Becton–Dickinson) at five sample points designated as weeks 0, 6, 17, 22 and 36 in relation to i/n with *C. abortus* (Groups 1–3 and sham control Group 4) or weeks −21, −11, 0, 5 and 19 in relation to s/c inoculation with *C. abortus* (Group 5). PBMC were purified using a standard protocol [12], but without a separate buffy-coat preparation stage. Instead, whole blood was diluted 1:2 in PBS and layered directly onto the proprietary density gradient medium (Lymphoprep 1.067 g/L, Axis Shield Diagnostics Ltd, Dundee, UK).

### Lymphocyte stimulation assays

PBMCs were adjusted to 2 × 10^6^ cells/mL in IMDM supplemented with 10% ΔH FBS, 2 mM l-glutamine, gentamicin (50 µg/mL), 100 IU/mL penicillin, 50 μg/mL streptomycin and 50 µM beta-mercaptoethanol (Sigma-Aldrich, Dorset, UK). One hundred microliters of cell suspension was added to U-bottom tissue culture plates (Nunc, Thermofisher Scientific, Renfrew, UK). To the cells, 100 µL of previously titrated UV-killed *C. abortus* antigen, HEp-2 control lysate, ConA (5 μg/mL), or medium alone in quadruplicate wells for each treatment. Duplicate assay plates were set up for collection of culture supernatant after 96 h of culture for cytokine analyses and 120 h of culture for cellular proliferation measurement.

### Cell proliferation assays

PBMCs were labelled with [methyl-^3^H]-thymidine (0.5 μCi/well) (Amersham Biosciences UK Ltd, Little Chalfont, Buckinghamshire, UK) for the last 18 h of a 120 h culture (as described in [12]). Cells were then harvested onto glass-fibre filters and radioactive signal measured using a 1450 MicroBeta TriLuxMicroplate Scintillation and Luminescence Counter (Perkin Elmer, Cambridge, UK). Data (treatment/animal) were collated and the counts per minute (cpm) values expressed as geometric means of quadruplicate wells.

### Cytokine ELISAs

IFN-γ measurement has been described previously [11]. Those data were re-analysed in the context of the additional immunological parameters described here. Interleukin (IL)-4 and IL-10 were measured in an indirect ELISA using antibody pairs (mAb clones CC313 and CC314 for IL-4; CC318 and CC320 for IL-10, Bio-Rad Antibody Laboratories, Oxford UK) and quantified using recombinant bovine (rbov) IL-4 of known concentration and recombinant ovine IL-10 of known biological activity [12, 17]. IL-17A was assayed and quantified using the bovine IL-17A ELISA kit (Kingfisher Biotech, Minneapolis, MN, USA) with rbov IL-17A standards, according to the manufacturer’s instructions; the kit cross-reactivity for ovine IL-17A has previously been reported [18].

### Statistical analyses

The basic summary statistics were compiled for each immunological parameter and formal statistical comparisons were based on linear mixed models (LMMs) with identity link function and Gaussian errors fitted by restricted maximum likelihood to rank-based inverse normal transformed data. Group, week and term accounting for the interaction between them were included as fixed-effect factors in the models, whereas animal ID was a random effect. Significance of the fixed effects was assessed by conditional F-tests. Post hoc pair-wise comparisons between groups and weeks used t-tests based on estimated marginal means from the LMM fits, with the corresponding *p*-values being adjusted for multiplicity using the Benjamini and Hochberg’s method [19]. Separated LMMs were fitted including outcome of pregnancy (lambing or abortion) and potential interactions with the other factors as additional fixed effects to determine if there were specific associations with the immune responses measured for groups 1, 2 and challenge control group 5. However, this was not possible for the highest i/n dose group (group 3) due to only one abortion taking place. All the statistical analyses were performed on the R system for statistical computing v3 [20]. Significance tests were assessed at the usual 5% significance level.

## Results

### Baseline immunological responses for assignment of experimental animal groups

**Figure 2:**
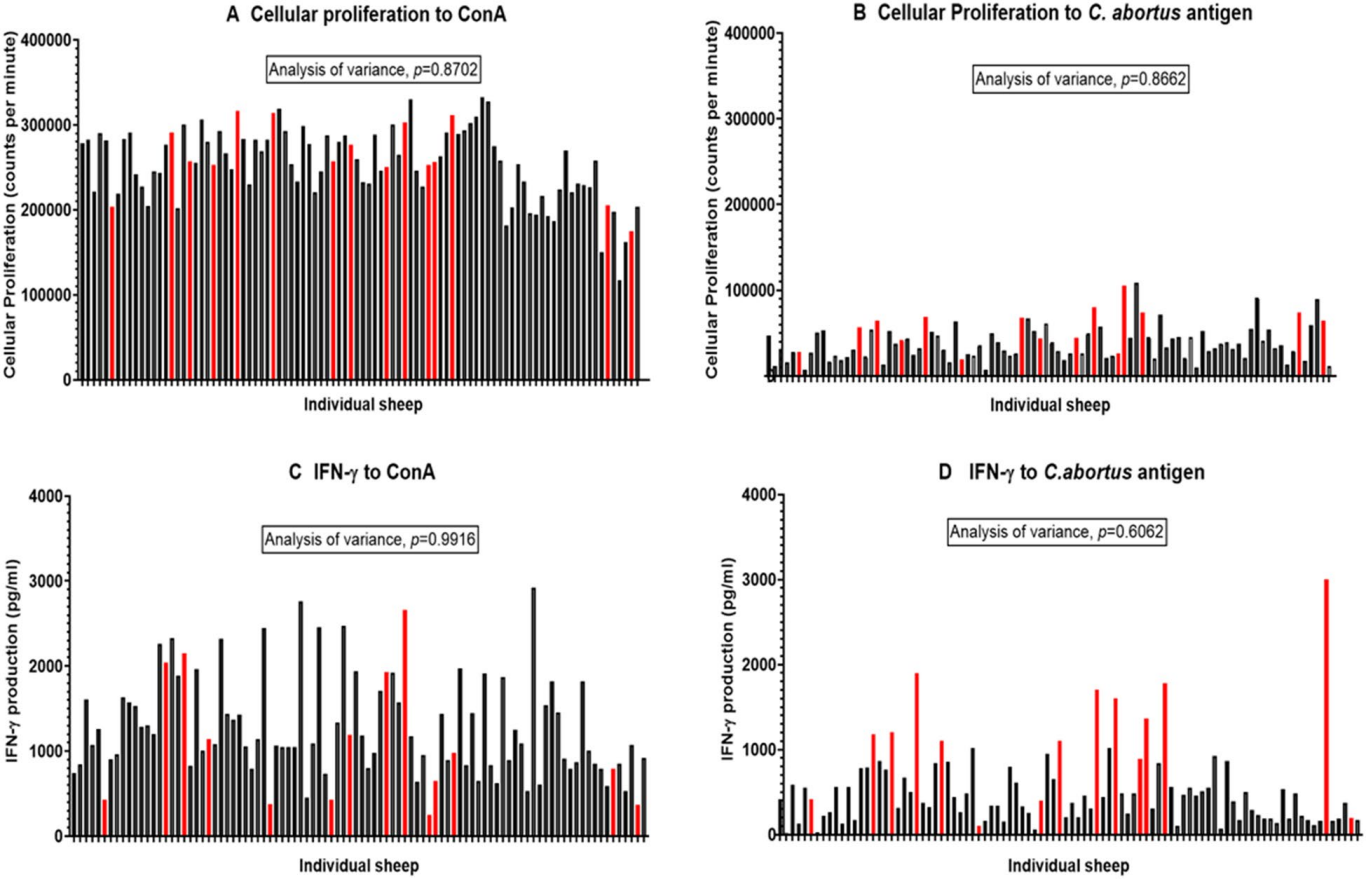
**Cellular immune responses of sheep used for selection and assignment of experimental groups.** Peripheral blood mononuclear cells from the 94 sheep were purified and set up in lymphocyte stimulation assays in vitro at week 0 with the mitogen Concanavalin A (ConA) and UV-inactivated *Chlamydia abortus* (*C. abortus*) antigen (as described in full in section “Lymphocyte stimulation assays”) with duplicate plates set up for the measurement of cellular proliferation (as described in full in section “Cell proliferation assays”) and culture supernatants (collected at 96 h). In brief, cellular proliferation was measured for the last 18 h of the 120 h culture, 0.5 microCurie/well was added and plates were harvested and data collated for individual animals as the geometric mean of quadruplicate values. Interferon-gamma (IFN-γ) production was measured in the culture supernates by a specific quantifiable ELISA (as previously described [11]). The data are displayed in bar graphs with individual animal data shown as single bars. Black bars represent sheep retained for the study and red bars represent sheep removed from the study. **A** Cellular proliferation to ConA. Where the x axis displays data for individual animals and the y axis shows the cellular proliferation data presented in counts per minute units. **B** Cellular proliferation to *C. abortus* antigen, where the x axis displays data for individual animals and the y axis shows the cellular proliferation data presented in counts per minute units. **C** IFN-γ production to ConA, where the x axis displays data for individual animals and the y axis the IFN-γ production units in picograms/millilitre. **D** IFN-γ production to *C. abortus* antigen, where the x axis displays data for individual animals and the y axis the IFN-γ production units in picograms/millilitre. The statistics displayed on each graph have been derived from Linear Mixed Modelling (LMM) as described in detail in section “Statistical analyses”.

The animals were assigned in the study on the basis of the criteria documented for cellular proliferation and IFN-γ production shown in Table 1. PBMC were screened for recall responses to *C. abortus* antigen and for stimulation with the T cell mitogen ConA to establish baselines assigned as week 0 prior to group allocation and inoculation, as shown in Figures 2A–D. Responses of each animal are represented by a single bar, with red bars indicating the animals excluded from the study based on a combination of the cut-off criteria listed in Table 1 (high background responses to antigen and/or low stimulation with ConA). The retained animals were then ranked by the magnitude of their PBMC to produce IFN-γ in response to ConA. Animals of “low, medium and high IFN-γ production capacity” were then systematically assigned to each experimental group. Once assigned, statistical comparisons of the groups revealed no statistically significant differences in their cellular responses to ConA, as determined by IFN-γ production (*p* = 0.9916) or cellular proliferation (*p* = 0.8702). Equally, the baseline responses to *C. abortus* antigen were consistent across the groups for both IFN-γ production (*p* = 0.6062) and cellular proliferation (*p* = 0.8662). These data served as a solid basis for determining antigen-specific cellular responses between the groups following inoculation with *C. abortus*.

### Summary of pregnancy outcomes

The five experimental groups gave rise to the following outcomes at parturition: group 1 (low dose) 6 lambed and 10 abortions (59% abortion rate); group 2 (medium dose) 6 lambed and 12 abortions (67% abortion rate); group 3 (high dose) 18 lambed and 1 abortion (5% abortion rate); group 4 (sham control) 8 lambed; and group 5 (s/c control) 3 lambed and 3 abortions (50% abortion rate). Abortions due to *C. abortus* were confirmed by microbiological assessments, as previously described [11].

### Cell-mediated immune responses

The PBMC responses to culture medium and the negative control antigen (HEp-2 cell lysate control) treatments served as set up controls for the cellular assays. The cellular proliferation and cytokine production of PBMC cultured in these conditions were all low or undetectable and are therefore not shown.

#### Cellular proliferation to ConA

The cellular proliferation to ConA was broadly consistent throughout the study for all the groups (*p* = 0.5364) with a geometric mean of 200 000 to 300 000 cpm (Additional files 1A–E). There is a significant effect of time (week *p* < 0.0001) with the proliferative response notionally changing over time, statistically significant peaking at week 6 (*p* < 0.0081). The lowest responses were observed at week 36 post i/n and week 19 s/c inoculation (*p* < 0.0333).

#### IFN-γ production to ConA

The capacity of PBMC to produce IFN-γ in response to ConA stimulation was also consistent between groups (*p* = 0.5778) with the magnitude of responses significantly lower at week 6 and 17 i/n (week -11 and week 0 for s/c group 5) compared to other weeks (*p* < 0.0292). There is a statistically significant effect of time (*p* < 0.0001) suggesting variability of the IFN-γ response but no interaction between group and time effects (*p* = 0.1444; Additional files 2A–E).

#### IL-4 production to ConA

The IL-4 production to ConA appears to gradually increase leading to week 22 i/n (*p* < 0.0001) but the patterns are consistent across the groups (*p* = 0.5065; Additional files 3A–E). There is greater variability of response across time for the sham control group 4 and s/c control group 5 that may be contributing to the significant interaction between group and time (*p* = 0.0316) with apparent evidence of divergent group responses over time.

#### IL-10 production to ConA

The IL-10 response to ConA across time was variable (*p* < 0.0001) generally dropping in magnitude to week 36 i/n. Groups 4 and 5 were particularly inconsistent and variable as shown by the large error bars (Additional files 4D–E) and again this may be contributing to the significant interaction between group and time (*p* = 0.0040).

#### IL-17A production to ConA

There is no statistically significant difference in IL-17A production to ConA between the groups (*p* = 0.6450) over the duration of the study. IL-17A production to ConA across groups peaked around week 6 (*p* < 0.495) and gradually dropping thereafter to week 36 i/n (*p* < 0.0672). The pattern of responses in groups 4 and 5 are less consistent over time contributing to the time effect (*p* < 0.0001; Additional files 5A–E). Groups 1–3 had similar patterns of responses over time and that contributed to the non-significant interaction effect (*p* = 0.0652).

### In vitro recall responses to *C. abortus* antigen

#### Cellular proliferation to C. abortus antigen

**Figure 3:**
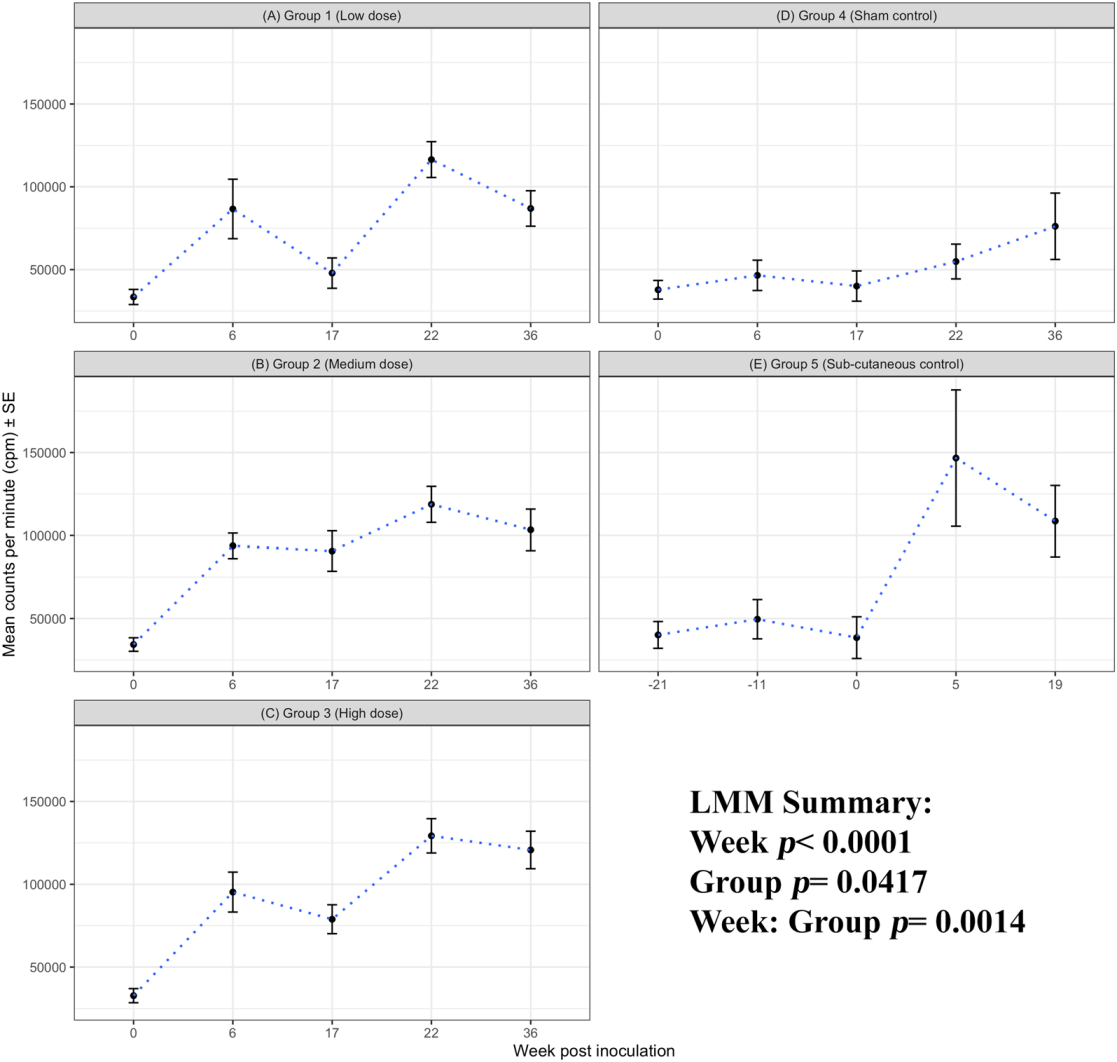
**Cellular proliferation to*C. abortus*antigen.** Peripheral blood mononuclear cells from the 67 sheep in the five experimental groups were purified from whole blood (as described in section “Lymphocyte stimulation assays”) on five occasions and set up in lymphocyte stimulation assays in vitro with the UV-inactivated *Chlamydia abortus* (*C. abortus*) antigen (set up as described in section “Lymphocyte stimulation assays”). One set of the duplicate plates were analysed for cellular proliferation (described in full detail, section “Cell proliferation assays”). In brief, cellular proliferation was measured for the last 18 h of the 120 h culture, 0.5 microCurie/well was added and plates were harvested and data collated for individual animals as the geometric mean of quadruplicate values. The datasets from each experimental group is presented in individual line graphs. The data points are the arithmetic mean values for each cellular bleed and the error bars represent the standard error of the mean (SEM). The x axis represents the weeks post inoculation with *C. abortus* (intranasal inoculation (i/n) Groups 1–3 with i/n sham control Group 4; and sub-cutaneous inoculation (s/c) Group 5). The week numbering for groups 1–4 are consistent in relation to i/n whereas group 5 is in relation to s/c. The y axis represents the arithmetic mean cellular proliferation in counts per minute. **A** Group 1 (low dose), **B** Group 2 (medium dose), **C** Group 3 (high dose), **D** Group 4 (sham control) and **E** Group 5 (sub-cutaneous control). The statistics summarised in the figure been derived from Linear Mixed Modelling (LMM) as described in detail in section “Statistical analyses”.

The background responses to chlamydial antigens were generally below 50 000 cpm and equivalent across all groups at week 0 prior to oronasal inoculation (*p* = 0.9916; Figure 2B). Analysis of the cellular proliferation to the chlamydial antigen by LMM reveals that responses significantly change over time across the groups (week, *p* < 0.0001, Figures 3A–E) for the entire study. This gives rise to clear patterns of responses to i/n group 1–3 and s/c infection group 5 (group *p* = 0.0417) when compared with the sham control group 4 (for all five cellular bleeds). The divergence of the responses between the groups over time gives a significant interaction effect (week: group, *p* = 0.0014).

There are specific changes in the magnitude of the cellular proliferation groups 1–3 vs group 4; and group 5 vs group 4 that coincide with post i/n and post s/c bleeds (week 6 i/n and week 22 i/n/week 0 s/c), respectively, when compared to the proceeding bleed (week 0 vs week 6 i/n, *p* < 0.0001; and week 0 vs week 5 s/c, *p* < 0.0001). This is a direct change in the response to *Chlamydia* infection and is statistically significant across all five experimental groups. This shows clear trends of elevated proliferation to i/n groups 1–3 vs group 4 at week 6, (*p* < 0.1908) or s/c *C. abortus* infection week 5 for group 5 vs group 4 (*p* = 0.0092).

At the pre-parturition bleed (i/n week 22 and s/c week 5), the magnitude of the proliferative responses in groups 1–3 plus 5 over the sham control group are highly statistically significant (*p* < 0.0092). This clear response in the i/n and s/c groups at week 22 i/n and week 5 s/c drops at the week 36 i/n (or week 19 s/c) post-parturition bleed.

#### Cytokine production to *C. abortus* antigen

As stated earlier, animals were allocated to groups on the basis of PBMC proliferation and IFN-γ production. Analyses of PBMC supernatants for IL-4, IL-10 and IL-17A also revealed no inter-group differences, consistent with the patterns observed for cellular proliferation and IFN-γ production at week 0 i/n (or week -21 s/c).

#### IFN-γ production

**Figure 4:**
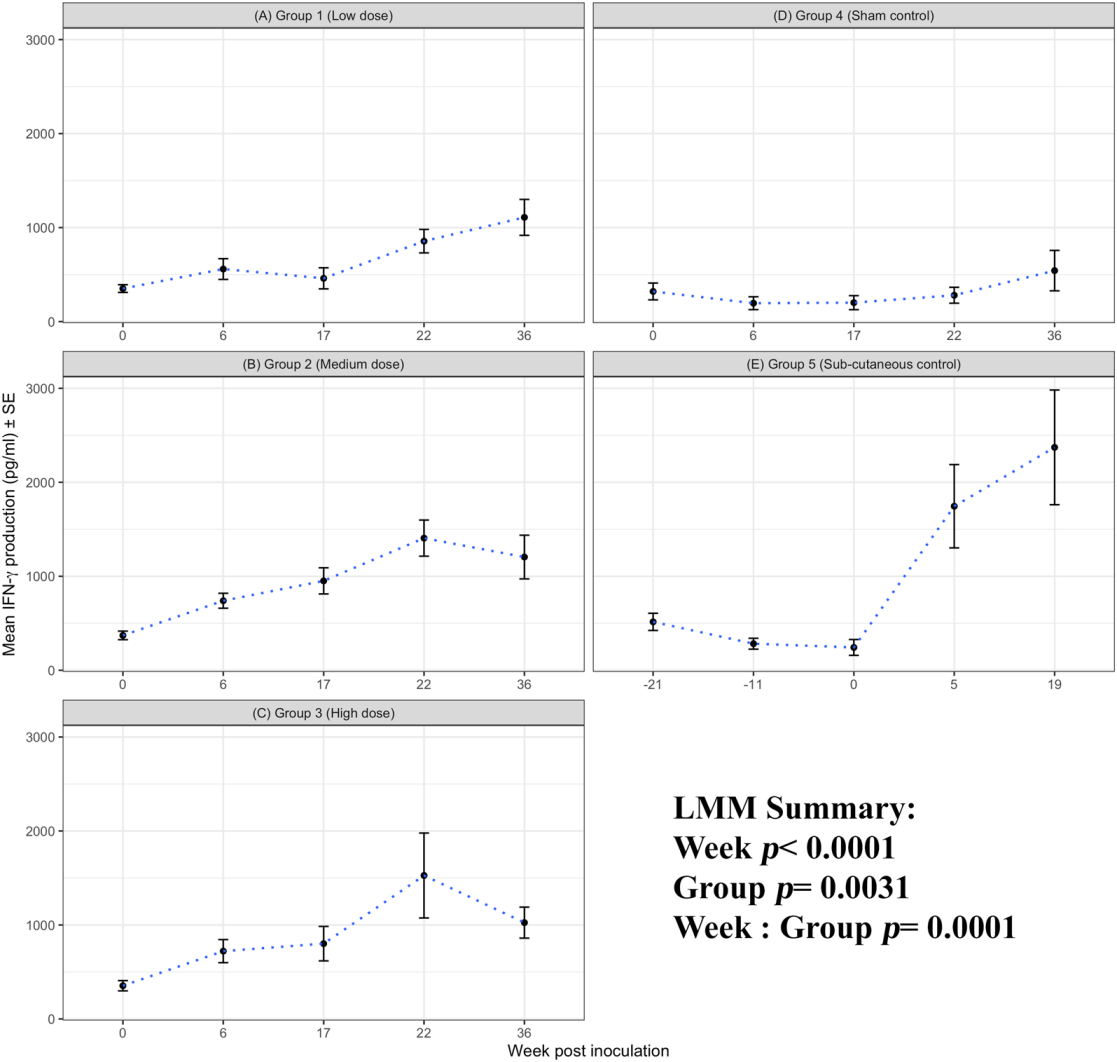
**IFN-γ production to*C. abortus*antigen.** Peripheral blood mononuclear cells from the 67 sheep in the five experimental groups were purified from whole blood (as described in section “Blood collection and peripheral blood mononuclear cell (PBMC) preparation”) on five occasions and set up in lymphocyte stimulation assays in vitro with the UV-inactivated *Chlamydia abortus* (*C. abortus*) antigen (set up as described in section “Lymphocyte stimulation assays”). One set of the duplicate plates were harvested for culture supernatants after 96 h and analysed for interferon-gamma (IFN-γ) production (as previously described [11]). The datasets from each experimental group is presented in individual line graphs (**A**–**E**). The data points are the arithmetic mean values for each cellular bleed and the error bars represent the standard error of the mean (SEM). The x axis represents the weeks post inoculation with *C. abortus* (intranasal inoculation (i/n) Groups 1–3 with i/n sham control Group 4; and sub-cutaneous inoculation (s/c) Group 5). The week numbering for groups 1–4 are consistent in relation to i/n whereas group 5 is in relation to s/c. The y axis represents the arithmetic mean IFN-γ production in picograms/millilitre concentration values. **A** Group 1 (low dose), **B** Group 2 (medium dose), **C** Group 3 (high dose), **D** Group 4 (sham control) and **E** Group 5 (sub-cutaneous control). The statistics summarised in the figure been derived from Linear Mixed Modelling (LMM) as described in detail in section “Statistical analyses”.

There is a meaningful effect of time on the antigen-driven IFN-γ response (week *p* < 0.0001), consistent with the cellular proliferation response. The i/n groups 1–3, s/c group 5 and sham control group 4 are significantly different (group *p* = 0.0031; Figures 4A–E) with these responses diverging over time (interaction effect week: group *p* = 0.0001).

The pattern of IFN-γ responses broadly follows that of the cellular proliferation with significant differences between the i/n groups 1–3 vs sham control group 4 (*p* < 0.0373) and s/c control group 5 v group 4 (*p* = 0.0267). The antigen-driven IFN-γ response increases following i/n from week 6 and peaks around week 22. There is variability in the magnitude of the responses between groups 1–3 over time but these differences are not consistent over time (*p* < 0.5147). The IFN-γ response significantly increases across the groups from week 17 to week 22 i/n and week 0 to week 5 s/c group 5 (*p* < 0.0001) corresponding to mid-pregnancy to the pre-abortion time period. All i/n groups 1–3 and s/c group 5 have significant IFN-γ production compared to the sham control group 4 (*p* < 0.0177). The IFN-γ response drops after parturition at week 36 but the level of responses are still elevated significantly over the sham control group 4 (*p* < 0.0478).

#### IL-4 production

The level of chlamydial antigen-driven IL-4 production was negligible across the five experimental groups and indistinguishable in magnitude from the HEp-2 negative control antigen (data not shown).

#### IL-10 production

**Figure 5:**
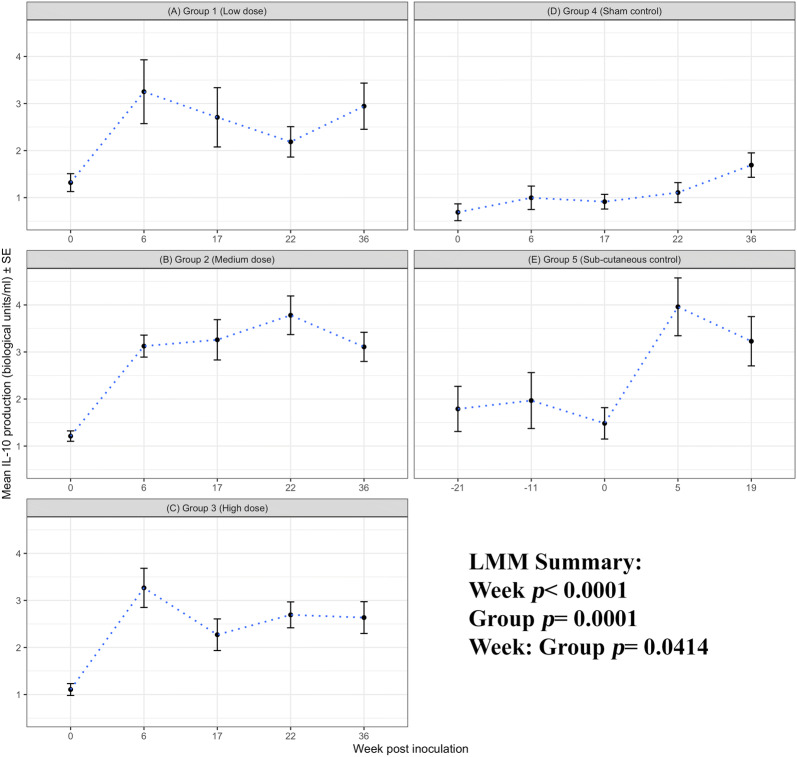
**IL-10 production to*C. abortus*antigen.** Peripheral blood mononuclear cells from the 67 sheep in the five experimental groups were purified from whole blood (as described in section “Blood collection and peripheral blood mononuclear cell (PBMC) preparation”) on five occasions and set up in lymphocyte stimulation assays in vitro with the UV-inactivated *Chlamydia abortus* (*C. abortus*) antigen (set up as described in section “Lymphocyte stimulation assays”). One set of the duplicate plates were harvested for culture supernatants after 96 h and analysed for interleukin (IL)-10 production (as described in section “Cytokine ELISAs”). The datasets from each experimental group are presented in individual line graphs (A–E). The data points are the arithmetic mean values for each cellular bleed and the error bars represent the standard error of the mean (SEM). The x axis represents the weeks post inoculation with *C. abortus* (intranasal inoculation (i/n) Groups 1–3 with i/n sham control Group 4; and sub-cutaneous inoculation (s/c) Group 5). The week numbering for groups 1–4 are consistent in relation to i/n whereas group 5 is in relation to s/c. The y axis represents the arithmetic mean IL-10 production in biological units/millilitre amounts. **A** Group 1 (low dose), **B** Group 2 (medium dose), **C** Group 3 (high dose), **D** Group 4 (sham control) and **E** Group 5 (sub-cutaneous control). The statistics summarised in the figure been derived from Linear Mixed Modelling (LMM) as described in detail in section “Statistical analyses”.

The magnitude of antigen-driven IL-10 responses were generally lower than responses simulated by ConA, However, there is a significant effect of week for chlamydial antigen-stimulated IL-10 production as determined by the LMM analyses (week, *p* < 0.0001). The IL-10 responses across the groups are significantly different (group, *p* = 0.0001; Figures 5A–E) and these responses to antigen diverge over time (week: group *p* = 0.0414). Each i/n group and the s/c group 5 have significantly different IL-10 responses when compared to the sham group 4 (*p* < 0.0032). There is a consistent significant increase in antigen-driven IL-10 in the time point following i/n *C. abortus* infection for groups 1–3 (week 0 vs week 6, *p* < 0.0001) and s/c for group 5 (week 0 vs week 5, *p* = 0.0056). This IL-10 response remained elevated in the infected groups through to the pre-abortion i/n week 22 and s/c week 5 over the sham control group 4.

#### IL-17A production

**Figure 6:**
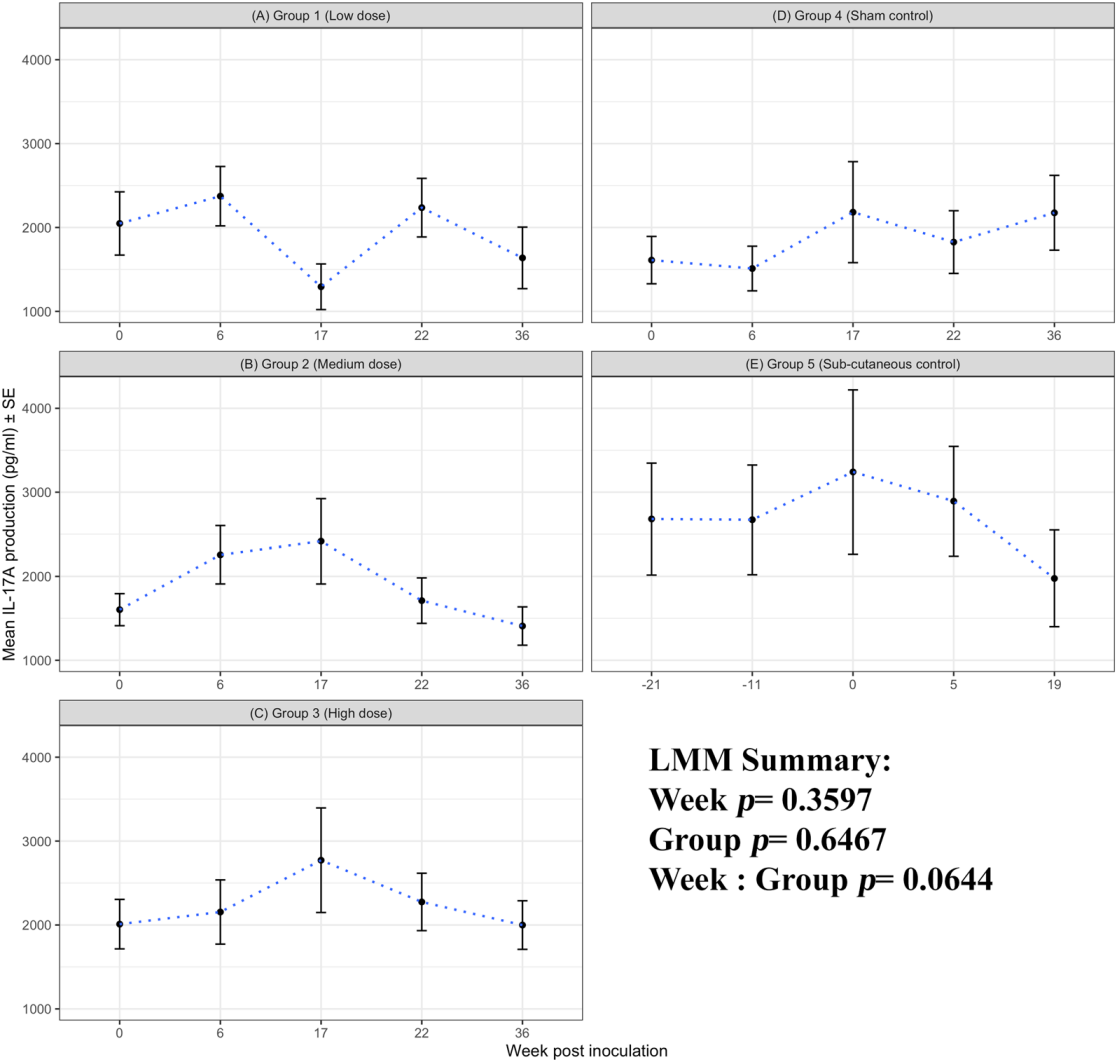
**IL-17A production to*C. abortus*antigen.** Peripheral blood mononuclear cells from the 67 sheep in the five experimental groups were purified from whole blood (as described in section “Blood collection and peripheral blood mononuclear cell (PBMC) preparation”) on five occasions and set up in lymphocyte stimulation assays in vitro with the UV-inactivated *Chlamydia abortus* (*C. abortus*) antigen (set up as described in section “Lymphocyte stimulation assays”). One set of the duplicate plates were harvested for culture supernatants after 96 h and analysed for interleukin (IL)-17A production (as described in section “Cytokine ELISAs”). The datasets from each experimental group are presented in individual line graphs (**A**–**E**). The data points are the arithmetic mean values for each cellular bleed and the error bars represent the standard error of the mean (SEM). The x axis represents the weeks post inoculation with *C. abortus* (intranasal inoculation (i/n) Groups 1–3 with i/n sham control Group 4; and sub-cutaneous inoculation (s/c) Group 5). The week numbering for groups 1–4 are consistent in relation to i/n whereas group 5 is in relation to s/c. The y axis represents the arithmetic mean IL-17A production in picograms/millilitre amounts. **A** Group 1 (low dose), **B** Group 2 (medium dose), **C** Group 3 (high dose), **D** Group 4 (sham control) and **E** Group 5 (sub-cutaneous control). The statistics summarised in the figure been derived from Linear Mixed Modelling (LMM) as described in detail in section “Statistical analyses”.

The magnitude of IL-17A production following exposure to chlamydial antigens (Figures 6A–E) was much lower than those to ConA (Additional file 5), irrespective of the infection group. In contrast to the IFN-γ and IL-10 production there was no sustained IL-17A response to i/n or s/c *C. abortus* infection (week, *p* = 0.3597). There was a lot of variability in the IL-17A response and any differences between groups were not sustained (group, *p* = 0.6467) nor did these responses concertedly diverge (week: group, *p* = 0.0644).

### Analysis of cellular immune correlates with outcome of pregnancy

**Figure 7:**
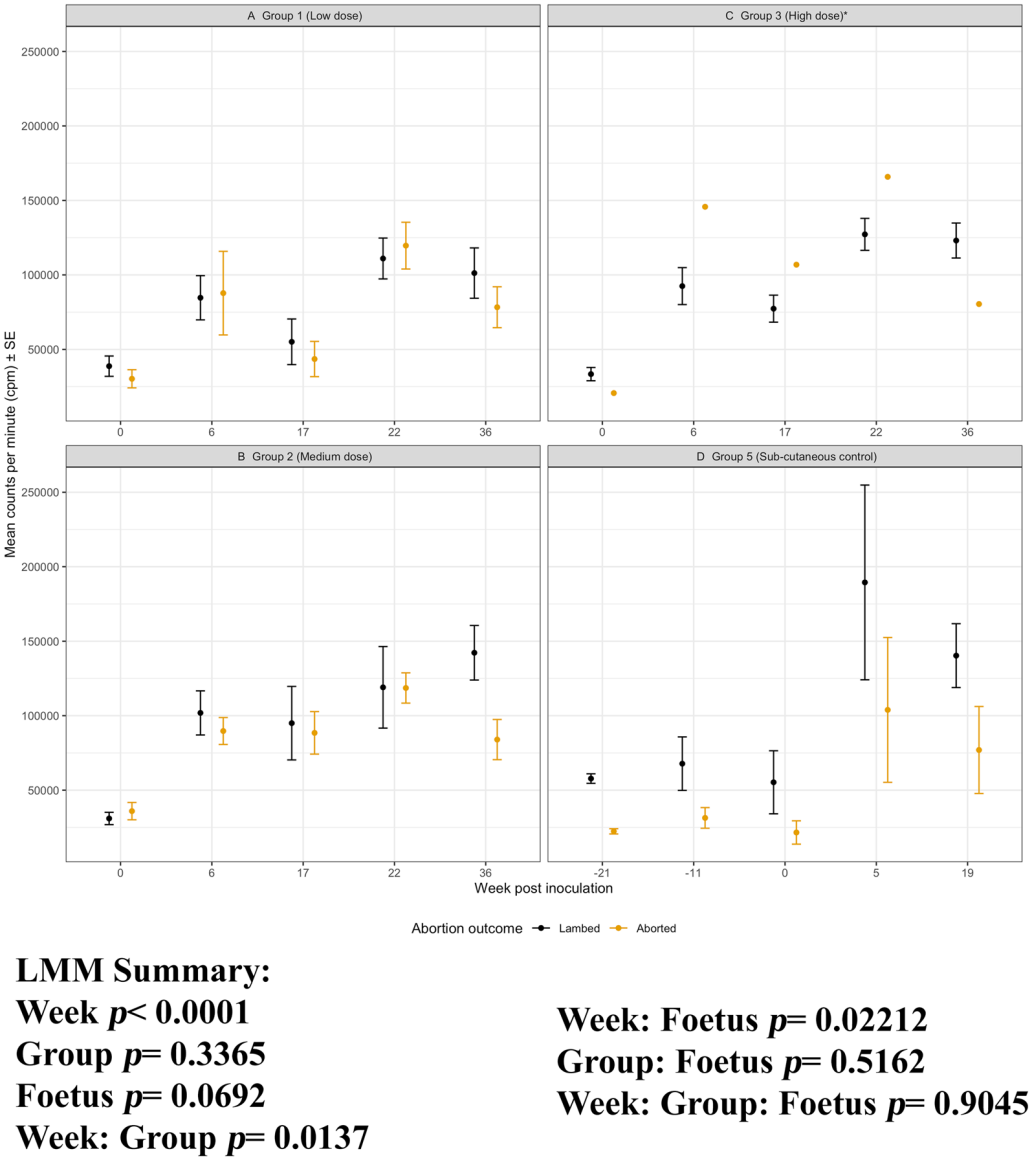
**Cellular proliferation to*C. abortus*antigen for Groups 1, 2, 3 and 5 separated by pregnancy outcome.** Peripheral blood mononuclear cells from the 67 sheep in the five experimental groups were purified from whole blood (as described in section “Blood collection and peripheral blood mononuclear cell (PBMC) preparation”) on five occasions and set up in lymphocyte stimulation assays in vitro with the UV-inactivated *Chlamydia abortus* (*C. abortus*) antigen (set up as described in section “Lymphocyte stimulation assays”). One set of the duplicate plates were analysed for cellular proliferation (described in full detail, section “Cell proliferation assays”). In brief, cellular proliferation was measured for the last 18 h of the 120 h culture, 0.5 microCurie/well was added and plates were harvested and data collated for individual animals as the geometric mean of quadruplicate values. The datasets from experimental group 1 (low dose), group 2 (medium dose), group 3 (high dose) and group 5 (sub-cutaneous control) are presented in graphs (**A**–**D**). In each graph the data has separated by outcome of pregnancy into aborted and lambed groups (as described in brief in section “Baseline immunological responses for assignment of experimental animal groups” or in full detail in Longbottom et al. [11]) represented yellow and black data points respectively. The data points are the arithmetic mean values for each cellular bleed and the error bars represent the standard error of the mean (SEM) except for group 3 where there is a single animal in the aborted group. The x axis represents the weeks post inoculation with *C. abortus* (intranasal inoculation (i/n) Groups 1 and 2 with sub-cutaneous inoculation (s/c) Group 5). The week numbering for groups 1, 2 and 3 are consistent in relation to i/n whereas group 5 is in relation to s/c. The y axis represents the arithmetic mean cellular proliferation in counts per minute units. (a) Group 1 (low dose), (b) Group 2 (medium dose) and (c) Group 3 (high dose and (d) Group 5 (sub-cutaneous control). The statistics summarised in the figure been derived from Linear Mixed Modelling (LMM) exclude analysis of group 3 (*) as described in detail in section “Statistical analyses” with a summary of estimates disclosed in Additional file 6).

**Figure 8:**
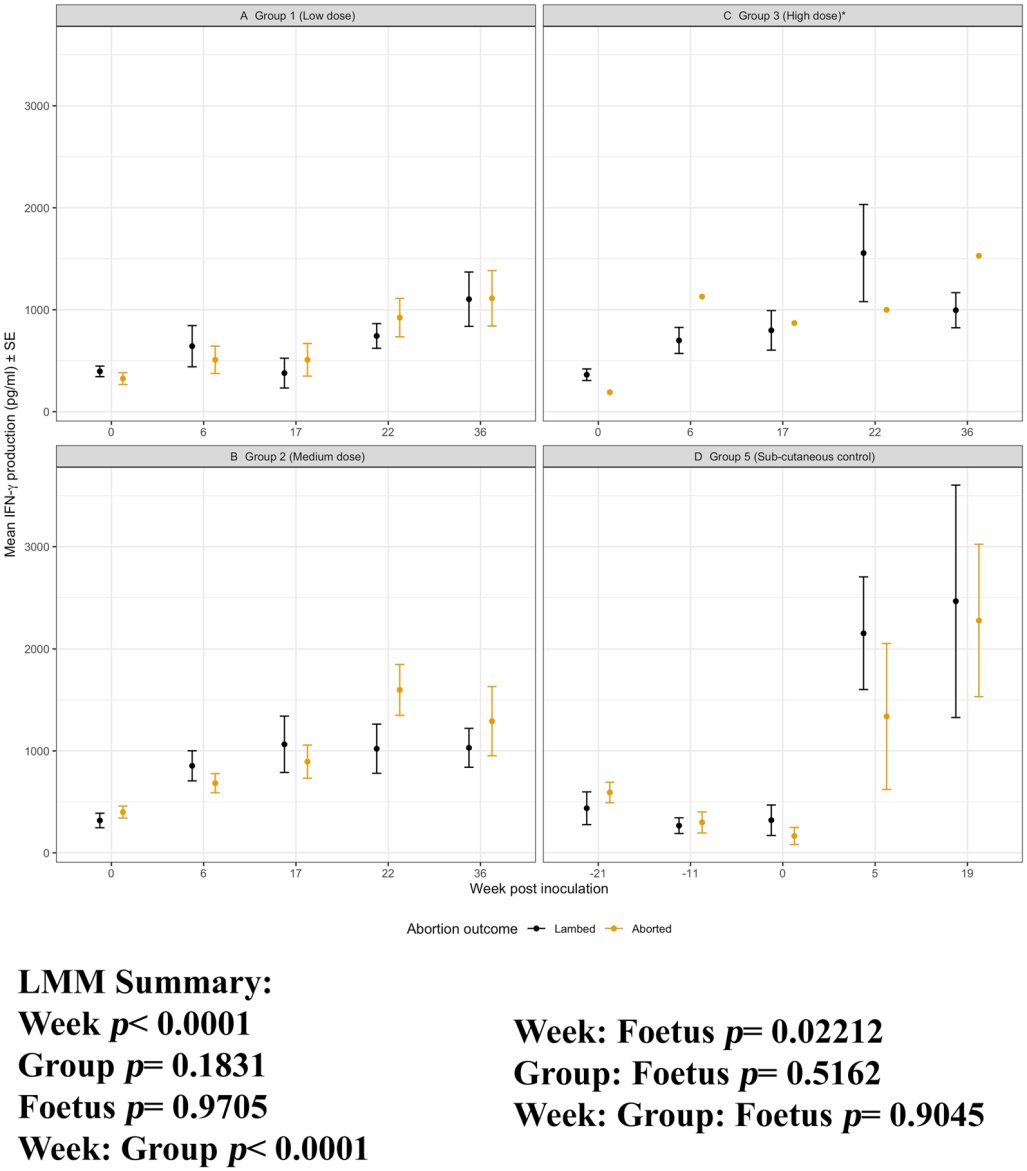
**IFN-γ production to*C. abortus*antigen for Groups 1, 2, 3 and 5 separated by pregnancy outcome.** Peripheral blood mononuclear cells from the 67 sheep in the five experimental groups were purified from whole blood (as described in section “Blood collection and peripheral blood mononuclear cell (PBMC) preparation”) on five occasions and set up in lymphocyte stimulation assays in vitro with the UV-inactivated *Chlamydia abortus* (*C. abortus*) antigen (set up as described in section “Lymphocyte stimulation assays”). One set of the duplicate plates were harvested for culture supernatants after 96 h and analysed for Interferon-gamma production (as previously described [8]). The datasets from experimental group 1 (low dose), group 2 (medium dose), group 3 (high dose) and group 5 (sub-cutaneous control) are presented in graphs **A**–**D**. In each graph the data has separated by outcome of pregnancy into aborted and lambed groups (as described in brief in section “Baseline immunological responses for assignment of experimental animal groups” or in full detail in Longbottom et al. [11]) represented yellow and black data points respectively. The data points are the arithmetic mean values for each cellular bleed and the error bars represent the standard error of the mean (SEM) except for group 3 where there is a single animal in the aborted group. The x axis represents the weeks post inoculation with *C. abortus* (intranasal inoculation (i/n) Groups 1, 2, and 3 with sub-cutaneous inoculation (s/c) Group 5). The week numbering for groups 1, 2 and 3 are consistent in relation to i/n whereas group 5 is in relation to s/c. The y axis represents the arithmetic mean IFN-γ production in pictograms/millilitre concentration values. **A** Group 1 (low dose), **B** Group 2 (medium dose), **C** Group 3 (high dose) and **D** Group 5 (sub-cutaneous control). The statistics summarised in the figure been derived from Linear Mixed Modelling (LMM) exclude analysis of group 3 (*) as described in detail in section “Statistical analyses” with a summary of estimates disclosed in Additional file 6).

**Figure 9:**
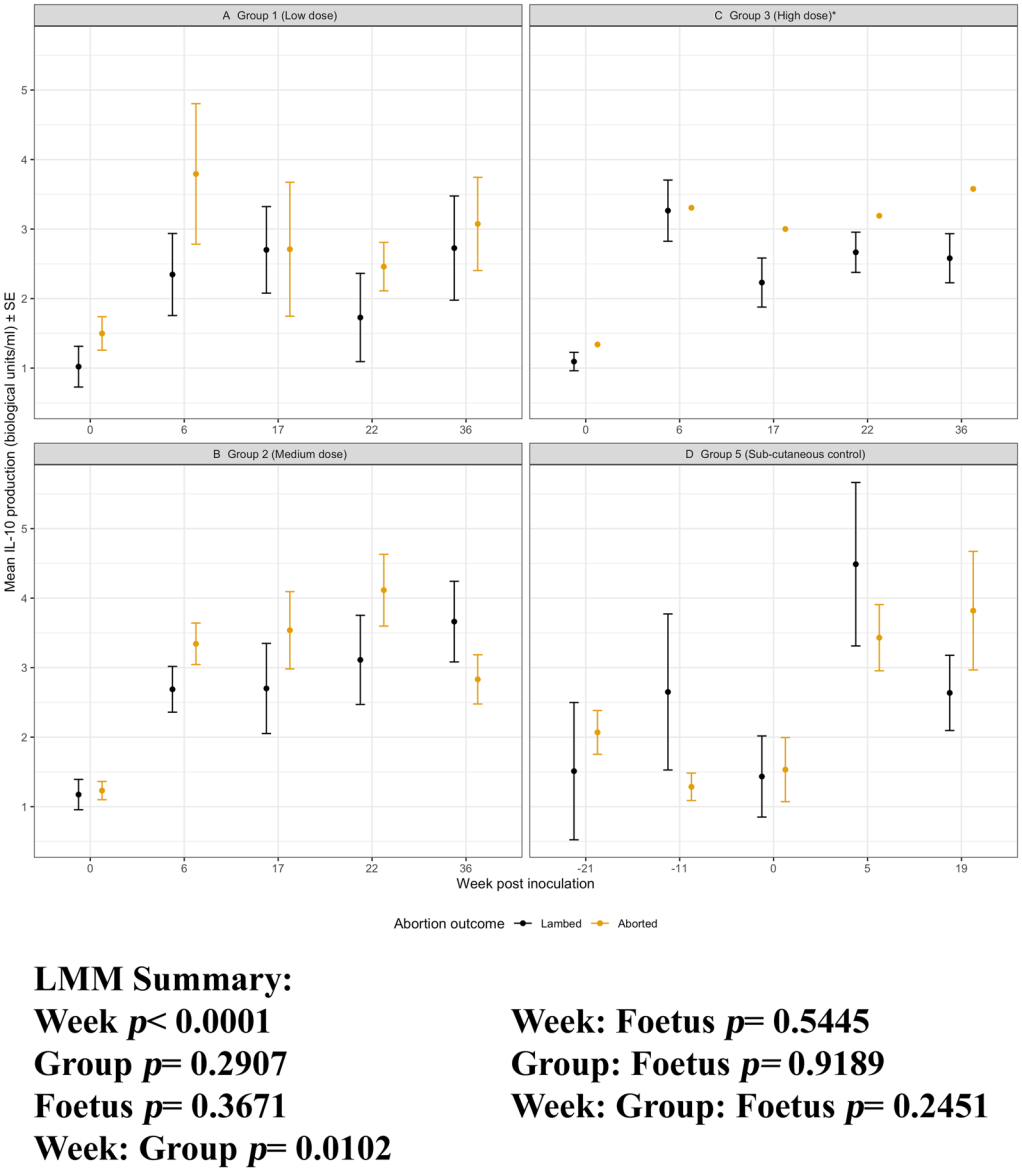
**IL-10 production to*C. abortus*antigen for Groups 1, 2, 3 and 5 separated by pregnancy outcome.** Peripheral blood mononuclear cells from the 67 sheep in the five experimental groups were purified from whole blood (as described in section “Blood collection and peripheral blood mononuclear cell (PBMC) preparation”) on five occasions and set up in lymphocyte stimulation assays in vitro with the UV-inactivated *Chlamydia abortus* (*C. abortus*) antigen (set up as described in section “Lymphocyte stimulation assays”). One set of the duplicate plates were harvested for culture supernatants after 96 h and analysed for interleukin (IL)-10 (as described in section “Cytokine ELISAs”). The datasets from experimental group 1 (low dose), group 2 (medium dose), group 3 (high dose) and group 5 (sub-cutaneous control) are presented in graphs (**A**–**D**). In each graph the data has separated by outcome of pregnancy into aborted and lambed groups (as described in brief in section “Baseline immunological responses for assignment of experimental animal groups” or in full detail in Longbottom et al. [11]) represented yellow and black data points respectively. The data points are the arithmetic mean IL-10 production values for each cellular bleed and the error bars represent the standard error of the mean (SEM) except for group 3 where there is a single animal in the aborted group. The x axis represents the weeks post inoculation with *C. abortus* (intranasal inoculation (i/n) Groups 1, 2 and 3 with sub-cutaneous inoculation (s/c) Group 5). The week numbering for groups 1, 2 and 3 are consistent in relation to i/n whereas group 5 is in relation to s/c. The y axis represents the arithmetic mean IL-10 production in biological units/millilitre values. **A** Group 1 (low dose), **B** Group 2 (medium dose), **C** Group 3 (high dose) and **D** Group 5 (sub-cutaneous control). The statistics summarised in the figure been derived from Linear Mixed Modelling (LMM) exclude analysis of group 3 (*) as described in detail in section “Statistical analyses” with a summary of estimates disclosed in Additional file 6).

**Figure 10:**
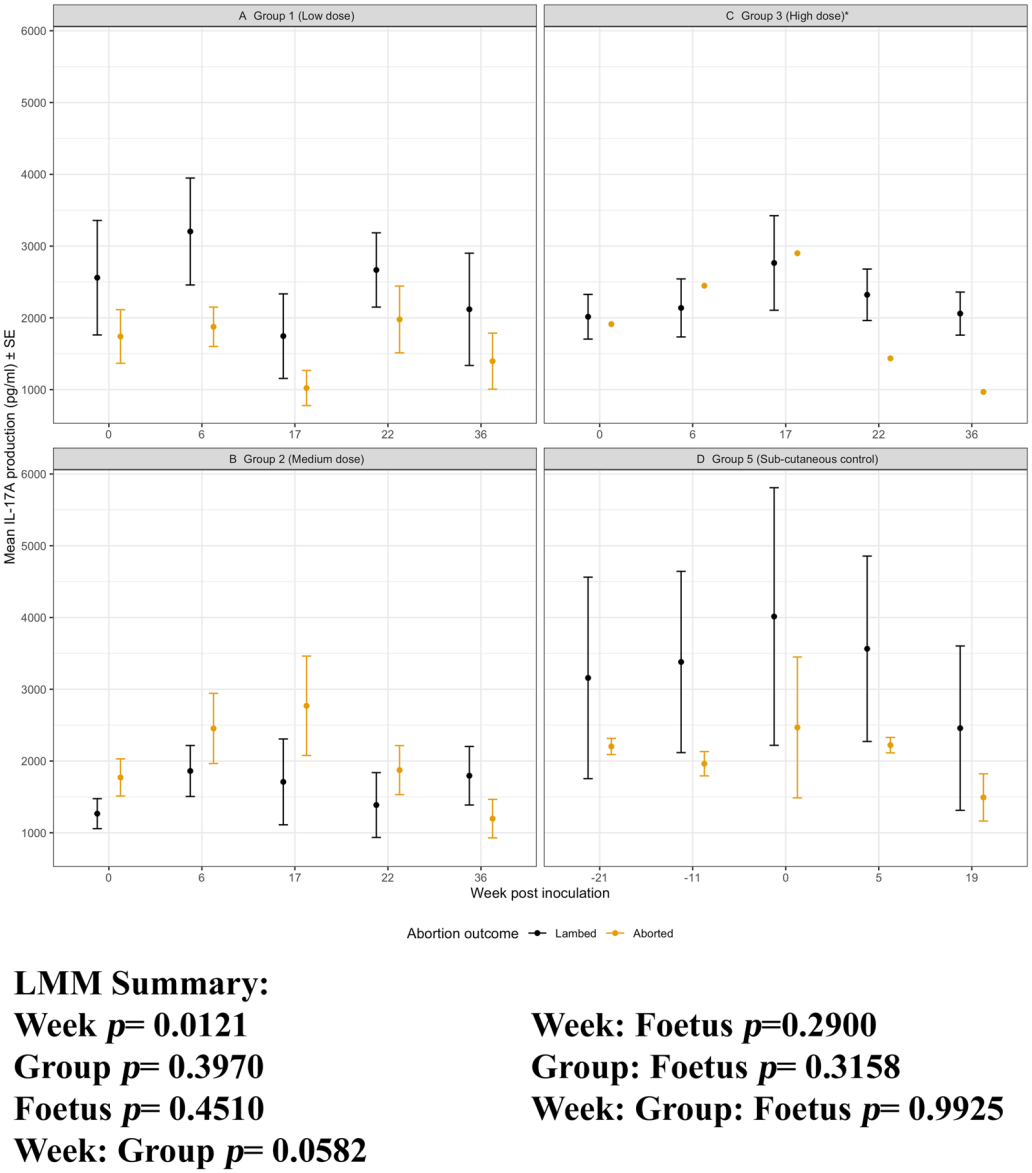
**IL-17A production to*C. abortus*antigen for Groups 1, 2, 3 and 5 separated by pregnancy outcome.** Peripheral blood mononuclear cells from the 67 sheep in the five experimental groups were purified from whole blood (as described in section “Blood collection and peripheral blood mononuclear cell (PBMC) preparation”) on five occasions and set up in lymphocyte stimulation assays in vitro with the UV-inactivated *Chlamydia abortus* (*C. abortus*) antigen (set up as described in section “Lymphocyte stimulation assays”). One set of the duplicate plates were harvested for culture supernatants after 96 h and analysed for interleukin (IL)-17A (as described in section “Cytokine ELISAs”). The datasets from experimental group 1 (low dose), group 2 (medium dose), group 3 (high dose) and group 5 (sub-cutaneous control) are presented in graphs (**A**–**D**). In each graph the data has separated by outcome of pregnancy into aborted and lambed groups (as described in brief in section “Baseline immunological responses for assignment of experimental animal groups” or in full detail in Longbottom et al. [11]) represented yellow and black data points respectively. The data points are the arithmetic mean IL-10 production values for each cellular bleed and the error bars represent the standard error of the mean (SEM) except for group 3 where there is a single animal in the aborted group. The x axis represents the weeks post inoculation with *C. abortus* (intranasal inoculation (i/n) Groups 1, 2 and 3 with sub-cutaneous inoculation (s/c) Group 5). The week numbering for groups 1, 2 and 3 are consistent in relation to i/n whereas group 5 is in relation to s/c. The y axis represents the arithmetic mean IL-17A production in picrogram/millilitre values. **A** Group 1 (low dose), **B** Group 2 (medium dose), **C** Group 3 (high dose) and **D** Group 5 (sub-cutaneous control). The statistics summarised in the figure been derived from Linear Mixed Modelling (LMM) as described in detail in section “Statistical analyses” with a summary of estimates disclosed in Additional file 6).

All four of the *C. abortus* i/n and s/c infection groups gave rise to both abortion and lambed outcomes and it was important to ascertain if the cellular immune responses stimulated by chlamydial antigen correlates with infection, disease and/or protection (from abortion). Group 3, the high dose i/n group gave unexpected results whereby there were 18 sheep that lambed and only one abortion. The results for group 3 are shown in Figures 7, 8,9, 10 for completeness of the datasets but the single abortion does not allow us to statistically associate responses by pregnancy outcome for the group. For the ConA responses (cellular proliferation, IFN-γ, IL-4, IL-10 and IL-17A production) there were no differences in the magnitude of the responses across the five experimental groups. The chlamydial antigen responses yielded different results at a whole group level for cellular proliferation, IFN-γ and IL-10 production and this warranted closer examination of the associations with pregnancy outcomes for groups 1, 2 and 5 (A full summary of the estimates from LMM is provided; Additional file 6).

For cellular proliferation, the specific responses in each group 1, 2, 3 and 5 split by outcome largely overlap (aborted groups vs lambed groups; Figures 7A, B and D). Across the groups (1, 2, 3 and 5), the trend suggests that responses from the lambed groups are generally higher than those from aborted groups but this is not statistically significant (foetus effect, *p* = 0.0692, group 3 is not part of this comparison). The responses from aborted groups and lambed groups have greater variability following i/n or s/c *C. abortus* infection as represented graphically at the sample point values with larger error bars (Figures 7A, B and D from week 6 i/n or week 5 s/c). Additionally, there are no associations between group response and pregnancy outcome (group: foetus, *p* = 0.5162). At the first bleed, post i/n for groups 1 and 2 and s/c group 5 (week 6 i/n and week 5 s/c, respectively), responses in the lambed groups are higher or equal to the responses in the aborted groups.

For IFN-γ production, there is largely overlapping responses between aborted and lambed groups across i/n low and medium dose groups and the s/c group 5 (Figures 8A, B and D). The responses between the different pregnancy outcomes vary over time. There are no specific differences between the groups 1, 2 and 5 (group, *p* = 0.1831) and no clear association between IFN-γ and outcome of pregnancy (foetus, *p* = 0.9705). At the first bleed post i/n for groups 1 and 2 and s/c group 5 the responses in lambed groups is higher than those that abort. The responses in group 3 differ, but as noted previously there is only one animal that aborted here.

For IL-10 production, the aborted and lambed group responses again largely overlap (Figures 9A, B and D). No clear differences by outcome across the groups 1, 2 and 5 (group: foetus, *p* = 0.9189) and no specific consistent pattern of responses associated with abortion or lambed groups (foetus, *p* = 0.3671). In the i/n groups 1 and 2, the magnitude of responses after infection (week 0, i/n) for three consecutive bleeds (weeks 6, 17 and 22) are consistently higher in aborted groups suggestive of an association with abortion outcome. The single animal that aborted in group 3 had elevated IL-10 responses when compared to the lambed group for three consecutive bleeds (weeks 17, 22 and 36). For completeness, the chlamydial antigen-driven IL-17A has also been analysed and presented by outcome of pregnancy. As observed in Figure 6, there are no distinctive patterns of responses emerging based on abortion or lambed outcome in groups 1, 2 and 5 (Figures 10A, B and D). The response is highly variable over time (week, *p* = 0.0121) but there are no clear differences between the same groups (Group, *p* = 0.3970) or pregnancy outcome (Foetus, *p* = 0.4510).

## Discussion

The development of improved methods for the prevention and control of OEA is dependent on the knowledge of immune responses in infected sheep and determining how these relate to disease and protection. This is complex for a disease such as OEA that involves a latent, subclinical phase in non-pregnant sheep and an active disease phase of bacterial multiplication in the placenta that precedes abortion [7]. The purpose of this study was to investigate cellular immune responses in the different stages of OEA with a view to identifying immunological signatures that correlate with infection, disease and/or protection. We focussed on the production of cytokines typically associated with the major CD4^+ve^ T_helper_ cell subsets (IFN-γ/T_h_1; IL-4/T_h_2; IL-17A/T_h_17; IL-10/T_reg_) and proliferation of PBMC in a model of intranasal infection of non-pregnant sheep with *C. abortus* that results in abortion in the subsequent pregnancy [11]. This is a very powerful model as it allows investigation of immune responses during latent chlamydial infection, a phase that has been very difficult to reproduce experimentally [11, 21].

One of the difficulties in the investigation of immune correlates in outbred species is animal-to-animal variation in immune responsiveness, exemplified by the inter-animal and intra-animal variability in mitogen-driven cellular immune responses in sheep in repeat analyses over time [12]. We therefore consider it important to ensure that experimental groups are balanced on immune responsiveness at the outset when comparisons between groups are integral to data interpretation. All of the animals in this study were initially identified as seronegative for *C. abortus* [11], but this does not account for cellular immunity. Using antigen-driven and mitogen-stimulated PBMC proliferation and IFN-γ production, we excluded sheep with a combined profile of the highest background responses to chlamydial EBs and lowest responses to ConA (Figure 2) and then allocated the sheep to groups that were statistically similar for those parameters. Interestingly, further analyses revealed no statistical differences in the baseline production of IL-4, IL-10 or IL-17A in response to antigen or mitogen between the groups (Figures 5, 6 and Additional files 3, 4, 5 respectively). This gave us further confidence that the groups were well-balanced for the subsequent immunological analyses in relation to experimental infection.

Infection resulted in an increase in antigen-specific cell proliferation, IFN-γ and IL-10 production by PBMC (Figures 3, 4, 5), but not IL-4 or IL-17A (Figure 6). The levels of IL-4 produced by antigen-stimulated PBMC were undetectable for the duration of the experiment, hence the data are not shown. The PBMC were capable of producing IL-4 as demonstrated by ConA stimulation (Additional file 3) indicating that IL-4 is not a major component of the ovine immune response to *C. abortus* irrespective of a mucosal or parenteral route of infection. The levels of IL-17A produced by PBMC in response to antigen varied across the duration of the experiment, but no pattern in relation to infection could be established (Figure 6). This is in contrast to production of IFN-γ and IL-10 that correlate with infection (Figures 4 and 5), suggesting that different CD4^+ve^ subsets may be activated by *C. abortus* infection in sheep.

Although PBMC proliferation, IFN-γ and IL-10 production correlate with infection, they do not correlate with protection. The three doses of intranasal infection resulted in different outcomes, with the Group 3 highest dose resulting in a very low abortion rate (one ewe) but this was not reflected in differences between the groups in any of the cellular immunological parameters measured (Figures 7, 8, 9, 10). This is despite a strong body of in vitro evidence showing that IFN-γ is a functional correlate of protection, capable of restricting the growth of *C. abortus* [7, 22]. There could be several reasons for this. We have used whole chlamydial antigen (UV-killed asynchronous mix of elementary bodies and reticulate bodies) in our recall assays, hence preferential protective cellular responses to antigens that are minor components of EBs may not be revealed under such assay conditions. Our cytokine assays are also conducted as a “snapshot” of the recall response in PBMC supernatants harvested 96 h after stimulation. We have previously shown that the relative expression of antigen-driven IFN-γ and IL-10 changes over that time period at both the molecular and protein level [13], so different collection time points could reveal different profiles. Such multi-sampling approaches are very difficult to conduct currently, but technological developments could make this more feasible in the future. We also need to be conscious of the fact that we are using PBMC as our immunological analyte while we are dealing with a pathogen that has an unknown site of latency and causes disease in the sheep placenta, where there is evidence of site-specific immune modulation [23].

Given the complexities of the disease coupled with the immunological parameters that we have been able to evaluate, it is extremely difficult to dissect out clear correlates of infection or protection. Taking all of these factors into account, systems biology approaches that encompass multiple analytes combined with bioinformatics are likely to be more informative for identification of immune correlates than single analyte approaches. Diverse technologies such as PCR, RNAseq, ELISA, multiplexing and flow cytometry are now being implemented to characterise immune responses for vaccine design [24]. These technologies are becoming more accessible within the veterinary field [25] and represent future opportunities to identify immunological correlates of infection (for diagnostics) and protection (for vaccines [26]).

## Supplementary information

**Additional file 1. Cellular proliferation to ConA.** Peripheral blood mononuclear cells from the 67 sheep in the five experimental groups were purified from whole blood (as described in section “Blood collection and peripheral blood mononuclear cell (PBMC) preparation”) on five occasions and set up in lymphocyte stimulation assays in vitro with the mitogen Concanavalin A (set up as described in section “Lymphocyte stimulation assays”). One set of the duplicate plates were analysed for cellular proliferation (described in full detail, section “Cell proliferation assays”). In brief, cellular proliferation was measured for the last 18 h of the 120 h culture, 0.5 microCurie/well was added and plates were harvested and data collated for individual animals as the geometric mean of quadruplicate values. The datasets from each experimental group is presented in individual line graphs. The data points are the arithmetic mean values for each cellular bleed and the error bars represent the standard error of the mean (SEM). The x axis represents the weeks post inoculation with *Chlamydia abortus* (intranasal inoculation (i/n) Groups 1–3 with i/n sham control Group 4; and sub-cutaneous inoculation (s/c) Group 5). The week numbering for groups 1**–**4 are consistent in relation to i/n whereas group 5 is in relation to s/c. The y axis represents the arithmetic mean cellular proliferation in counts per minute. (A) Group 1 (low dose), (B) Group 2 (medium dose), (C) Group 3 (high dose), (D) Group 4 (sham control) and (E) Group 5 (sub-cutaneous control). The statistics summarised in the figure been derived from Linear Mixed Modelling (LMM) as described in detail in section “Statistical analyses”.

**Additional file 2. IFN-γ production to ConA.** Peripheral blood mononuclear cells from the 67 sheep in the five experimental groups were purified from whole blood (as described in section “Blood collection and peripheral blood mononuclear cell (PBMC) preparation”) on five occasions and set up in lymphocyte stimulation assays in vitro with the mitogen Concanavalin A (set up as described in section “Lymphocyte stimulation assays”). One set of the duplicate plates were harvested for culture supernatants after 96 h and analysed for Interferon-gamma (IFN-γ) production (as previously described [11]). The datasets from each experimental group is presented in individual line graphs. The data points are the arithmetic mean values for each cellular bleed and the error bars represent the standard error of the mean (SEM). The x axis represents the weeks post inoculation with *Chlamydia abortus* (intranasal inoculation (i/n) Groups 1–3 with i/n sham control Group 4; and sub-cutaneous inoculation (s/c) Group 5). The week numbering for groups 1–4 are consistent in relation to i/n whereas group 5 is in relation to s/c. The y axis represents the arithmetic mean IFN-γ production in picograms/millilitre concentration values. (A) Group 1 (low dose), (B) Group 2 (medium dose), (C) Group 3 (high dose), (D) Group 4 (sham control) and (E) Group 5 (sub-cutaneous control). The statistics summarised in the figure been derived from Linear Mixed Modelling (LMM) as described in detail in section “Statistical analyses”.

**Additional file 3. IL-4 production to ConA.** Peripheral blood mononuclear cells from the 67 sheep in the five experimental groups were purified from whole blood (as described in section “Blood collection and peripheral blood mononuclear cell (PBMC) preparation”) on five occasions and set up in lymphocyte stimulation assays in vitro with the mitogen Concanavalin A (ConA) (set up as described in section “Lymphocyte stimulation assays”). One set of the duplicate plates were harvested for culture supernatants after 96 h and analysed for interleukin (IL)-4 production (as described in section “Cytokine ELISAs”). The datasets from each experimental group are presented in individual line graphs (A–E). The data points are the arithmetic mean values for each cellular bleed and the error bars represent the standard error of the mean (SEM). The x axis represents the weeks post inoculation with *Chlamydia abortus* (intranasal inoculation (i/n) Groups 1**–**3 with i/n sham control Group 4; and sub-cutaneous inoculation (s/c) Group 5). The week numbering for groups 1**–**4 are consistent in relation to i/n whereas group 5 is in relation to s/c. The y axis represents the arithmetic mean IL-4 production in picogram/millilitre concentrations. (A) Group 1 (low dose), (B) Group 2 (medium dose), (C) Group 3 (high dose), (D) Group 4 (sham control) and (E) Group 5 (sub-cutaneous control). The statistics summarised in the figure been derived from Linear Mixed Modelling (LMM) as described in detail in section “Statistical analyses”.

**Additional file 4. IL-10 production to ConA.** Peripheral blood mononuclear cells from the 67 sheep in the five experimental groups were purified from whole blood (as described in section “Blood collection and peripheral blood mononuclear cell (PBMC) preparation”) on five occasions and set up in lymphocyte stimulation assays in vitro with the mitogen Concanavalin A (ConA) (set up as described in section “Lymphocyte stimulation assays”). One set of the duplicate plates were harvested for culture supernatants after 96 h and analysed for interleukin (IL)-10 production (as described in section “Cytokine ELISAs”). The datasets from each experimental group are presented in individual line graphs (A**–**E). The data points are the arithmetic mean values for each cellular bleed and the error bars represent the standard error of the mean (SEM). The x axis represents the weeks post inoculation with *Chlamydia abortus* (intranasal inoculation (i/n) Groups 1**–**3 with i/n sham control Group 4; and sub-cutaneous inoculation (s/c) Group 5). The week numbering for groups 1**–**4 are consistent in relation to i/n whereas group 5 is in relation to s/c. The y axis represents the arithmetic mean IL-10 production in biological units/millilitre amounts. (A) Group 1 (low dose), (B) Group 2 (medium dose), (C) Group 3 (high dose), (D) Group 4 (sham control) and (E) Group 5 (sub-cutaneous control). The statistics summarised in the figure been derived from Linear Mixed Modelling (LMM) as described in detail in section “Statistical analyses”.

**Additional file 5. IL-17A production to ConA.** Peripheral blood mononuclear cells from the 67 sheep in the five experimental groups were purified from whole blood (as described in section “Blood collection and peripheral blood mononuclear cell (PBMC) preparation”) on five occasions and set up in lymphocyte stimulation assays in vitro with the mitogen Concanavalin A (ConA) (set up as described in section “Lymphocyte stimulation assays”). One set of the duplicate plates were harvested for culture supernatants after 96 h and analysed for interleukin (IL)-17A production (as described in section “Cytokine ELISAs”). The datasets from each experimental group are presented in individual line graphs (A–E). The data points are the arithmetic mean values for each cellular bleed and the error bars represent the standard error of the mean (SEM). The x axis represents the weeks post inoculation with *Chlamydia abortus* (intranasal inoculation (i/n) Groups 1–3 with i/n sham control Group 4; and sub-cutaneous inoculation (s/c) Group 5). The week numbering for groups 1–4 are consistent in relation to i/n whereas group 5 is in relation to s/c. The y axis represents the arithmetic mean IL-17A production in picogram/millilitre concentrations. (A) Group 1 (low dose), (B) Group 2 (medium dose), (C) Group 3 (high dose), (D) Group 4 (sham control) and (E) Group 5 (sub-cutaneous control). The statistics summarised in the figure been derived from Linear Mixed Modelling (LMM) as described in detail in section “Statistical analyses”.

**Additional file 6. Summary of estimates from linear mixed modelling of the cellular*Chlamydia abortus*antigen-driven cellular proliferation, interferon-gamma production, interleukin (IL)-10 and IL-17A production in relation to pregnancy outcome.** Here are the analysis of variance tables from the linear mixed model (LMM) fits relating to each immunological parameter graphically represented in the section “Analysis of cellular immune correlates with outcome of pregnancy”. The tables refer to (A) estimates of the statistical significance of the fixed-effects terms of the models, including week, treatment group, pregnancy outcome (term called Foetus: either lambed or aborted) and its potential 2-level and 3-level interactions; and (B) post hoc comparisons between groups for each pregnancy outcome from the model fits. Note that the comparisons are made between groups 1 (low dose), group 2 (medium dose), and the challenge control group 5 (s/c control) only as explained in the section “Statistical analyses”.

## Data Availability

All datasets are presented in the paper or additional files supporting the manuscript.

## Abbreviations

*C. abortus*: *Chlamydia abortus*
ConA: Concanavalin A
cpm: counts per minute
dg: days of gestation
DIVA: Differentiating Infected from Vaccinated Animals
EB: elementary body
EAE: enzootic abortion of ewes
FBS: fetal bovine serum
ΔH: heat-inactivated
IFU: inclusion forming units
i/n: inoculated intranasally
IFN-γ: Interferon-gamma
IL: Interleukin
IMDM: Iscove’s Modified Dulbecco’s Medium
LMMs: linear mixed models
OEA: Ovine enzootic abortion
PBMC: peripheral blood mononuclear cells
RB: reticulate body
s/c: subcutaneously
T_h_: T_helper_
SPG: sucrose-phosphate-glutamate buffer
