# Supplementary figures and images for: Defining immune correlates during latent and active chlamydial infection in sheep
Source: Vet Res. 2020 Jun 1;51:75. doi: 10.1186/s13567-020-00798-6 (PMC7268686; doi:10.1186/s13567-020-00798-6)

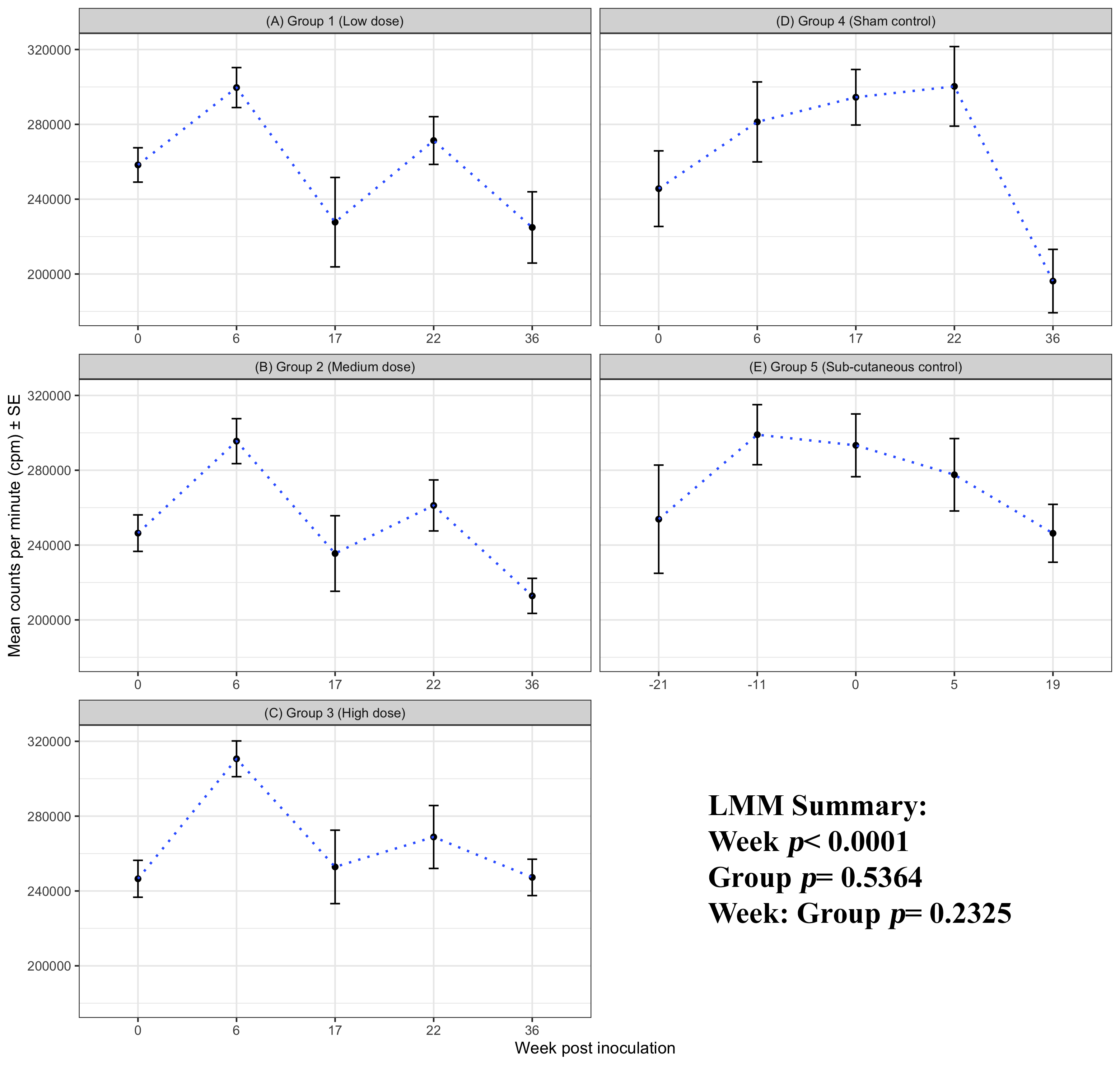

Supplement: Supplementary file 1 — Additional file 1. Cellular proliferation to ConA. Peripheral blood mononuclear cells from the 67 sheep in the five experimental groups were purified from whole blood (as described in section “Blood collection and peripheral blood mononuclear cell (PBMC) preparation”) on five occasions and set up in lymphocyte stimulation assays in vitro with the mitogen Concanavalin A (set up as described in section “Lymphocyte stimulation assays”). One set of the duplicate plates were analysed for cellular proliferation (described in full detail, section “Cell proliferation assays”). In brief, cellular proliferation was measured for the last 18 h of the 120 h culture, 0.5 microCurie/well was added and plates were harvested and data collated for individual animals as the geometric mean of quadruplicate values. The datasets from each experimental group is presented in individual line graphs. The data points are the arithmetic mean values for each cellular bleed and the error bars represent the standard error of the mean (SEM). The x axis represents the weeks post inoculation with Chlamydia abortus (intranasal inoculation (i/n) Groups 1–3 with i/n sham control Group 4; and sub-cutaneous inoculation (s/c) Group 5). The week numbering for groups 1–4 are consistent in relation to i/n whereas group 5 is in relation to s/c. The y axis represents the arithmetic mean cellular proliferation in counts per minute. (A) Group 1 (low dose), (B) Group 2 (medium dose), (C) Group 3 (high dose), (D) Group 4 (sham control) and (E) Group 5 (sub-cutaneous control). The statistics summarised in the figure been derived from Linear Mixed Modelling (LMM) as described in detail in section “Statistical analyses”. [file 13567_2020_798_MOESM1_ESM.tif]

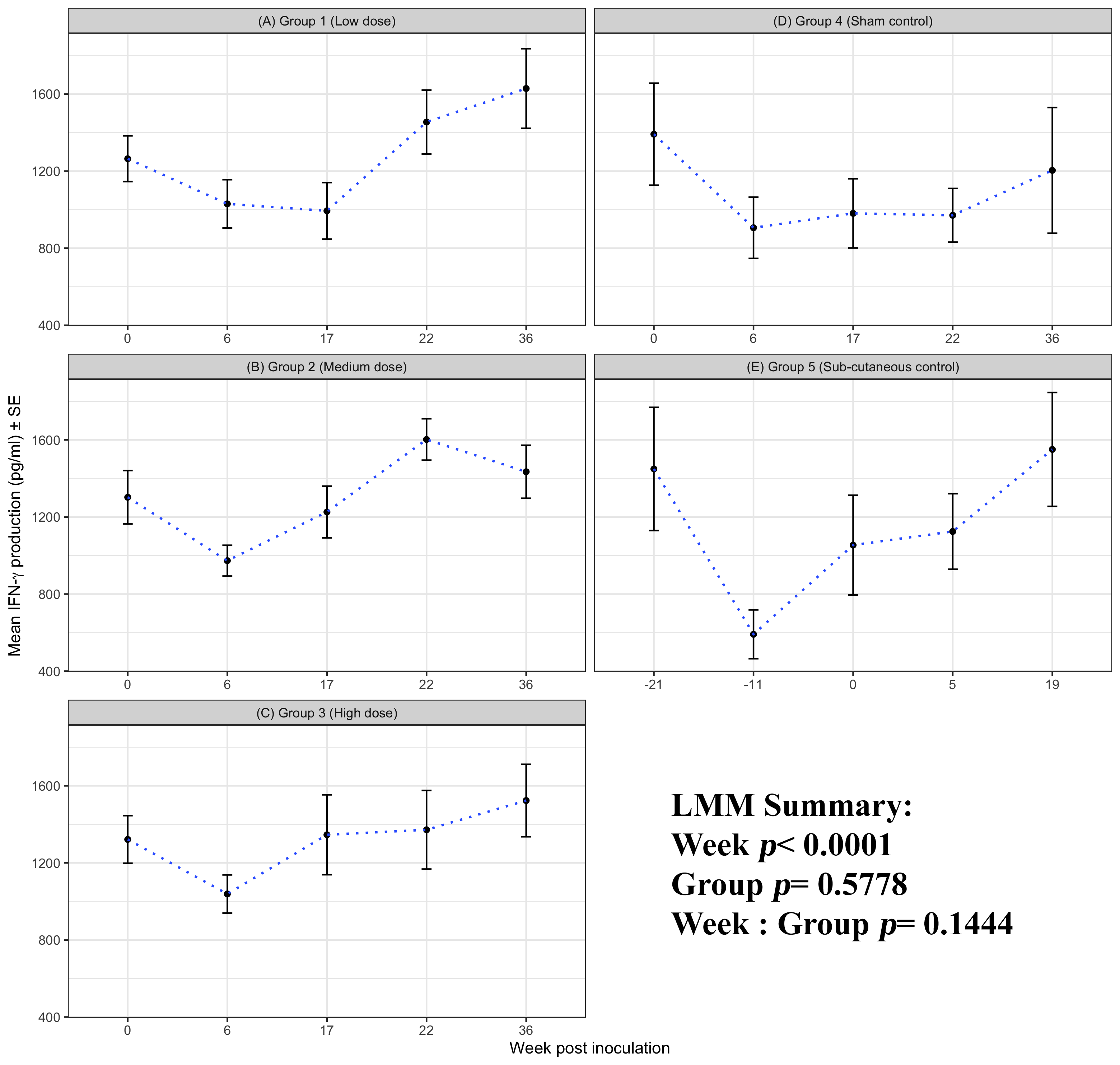

Supplement: Supplementary file 2 — Additional file 2. IFN-γ production to ConA. Peripheral blood mononuclear cells from the 67 sheep in the five experimental groups were purified from whole blood (as described in section “Blood collection and peripheral blood mononuclear cell (PBMC) preparation”) on five occasions and set up in lymphocyte stimulation assays in vitro with the mitogen Concanavalin A (set up as described in section “Lymphocyte stimulation assays”). One set of the duplicate plates were harvested for culture supernatants after 96 h and analysed for Interferon-gamma (IFN-γ) production (as previously described [11]). The datasets from each experimental group is presented in individual line graphs. The data points are the arithmetic mean values for each cellular bleed and the error bars represent the standard error of the mean (SEM). The x axis represents the weeks post inoculation with Chlamydia abortus (intranasal inoculation (i/n) Groups 1–3 with i/n sham control Group 4; and sub-cutaneous inoculation (s/c) Group 5). The week numbering for groups 1–4 are consistent in relation to i/n whereas group 5 is in relation to s/c. The y axis represents the arithmetic mean IFN-γ production in picograms/millilitre concentration values. (A) Group 1 (low dose), (B) Group 2 (medium dose), (C) Group 3 (high dose), (D) Group 4 (sham control) and (E) Group 5 (sub-cutaneous control). The statistics summarised in the figure been derived from Linear Mixed Modelling (LMM) as described in detail in section “Statistical analyses”. [file 13567_2020_798_MOESM2_ESM.tif]

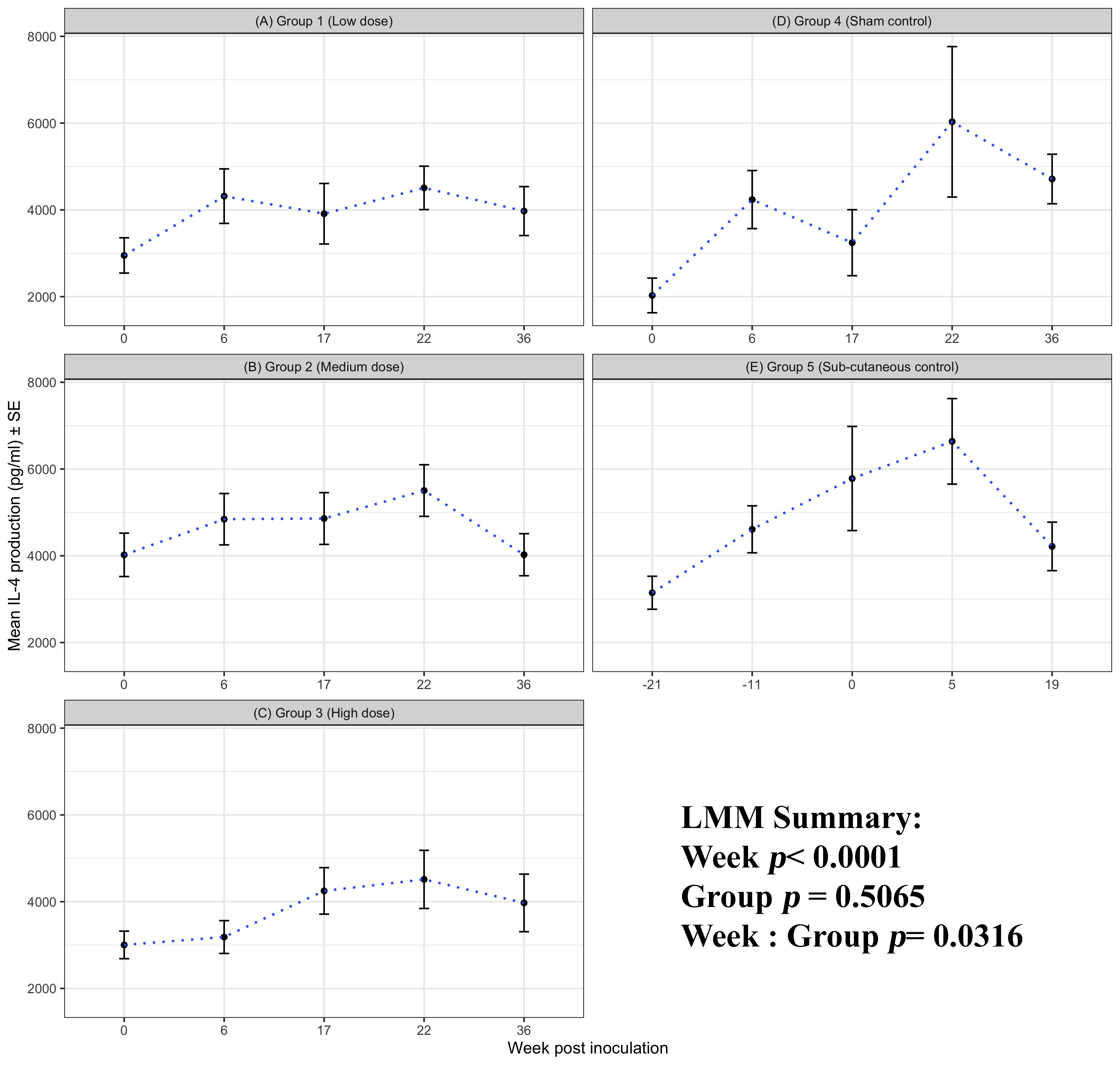

Supplement: Supplementary file 3 — Additional file 3. IL-4 production to ConA. Peripheral blood mononuclear cells from the 67 sheep in the five experimental groups were purified from whole blood (as described in section “Blood collection and peripheral blood mononuclear cell (PBMC) preparation”) on five occasions and set up in lymphocyte stimulation assays in vitro with the mitogen Concanavalin A (ConA) (set up as described in section “Lymphocyte stimulation assays”). One set of the duplicate plates were harvested for culture supernatants after 96 h and analysed for interleukin (IL)-4 production (as described in section “Cytokine ELISAs”). The datasets from each experimental group are presented in individual line graphs (A–E). The data points are the arithmetic mean values for each cellular bleed and the error bars represent the standard error of the mean (SEM). The x axis represents the weeks post inoculation with Chlamydia abortus (intranasal inoculation (i/n) Groups 1–3 with i/n sham control Group 4; and sub-cutaneous inoculation (s/c) Group 5). The week numbering for groups 1–4 are consistent in relation to i/n whereas group 5 is in relation to s/c. The y axis represents the arithmetic mean IL-4 production in picogram/millilitre concentrations. (A) Group 1 (low dose), (B) Group 2 (medium dose), (C) Group 3 (high dose), (D) Group 4 (sham control) and (E) Group 5 (sub-cutaneous control). The statistics summarised in the figure been derived from Linear Mixed Modelling (LMM) as described in detail in section “Statistical analyses”. [file 13567_2020_798_MOESM3_ESM.tif]

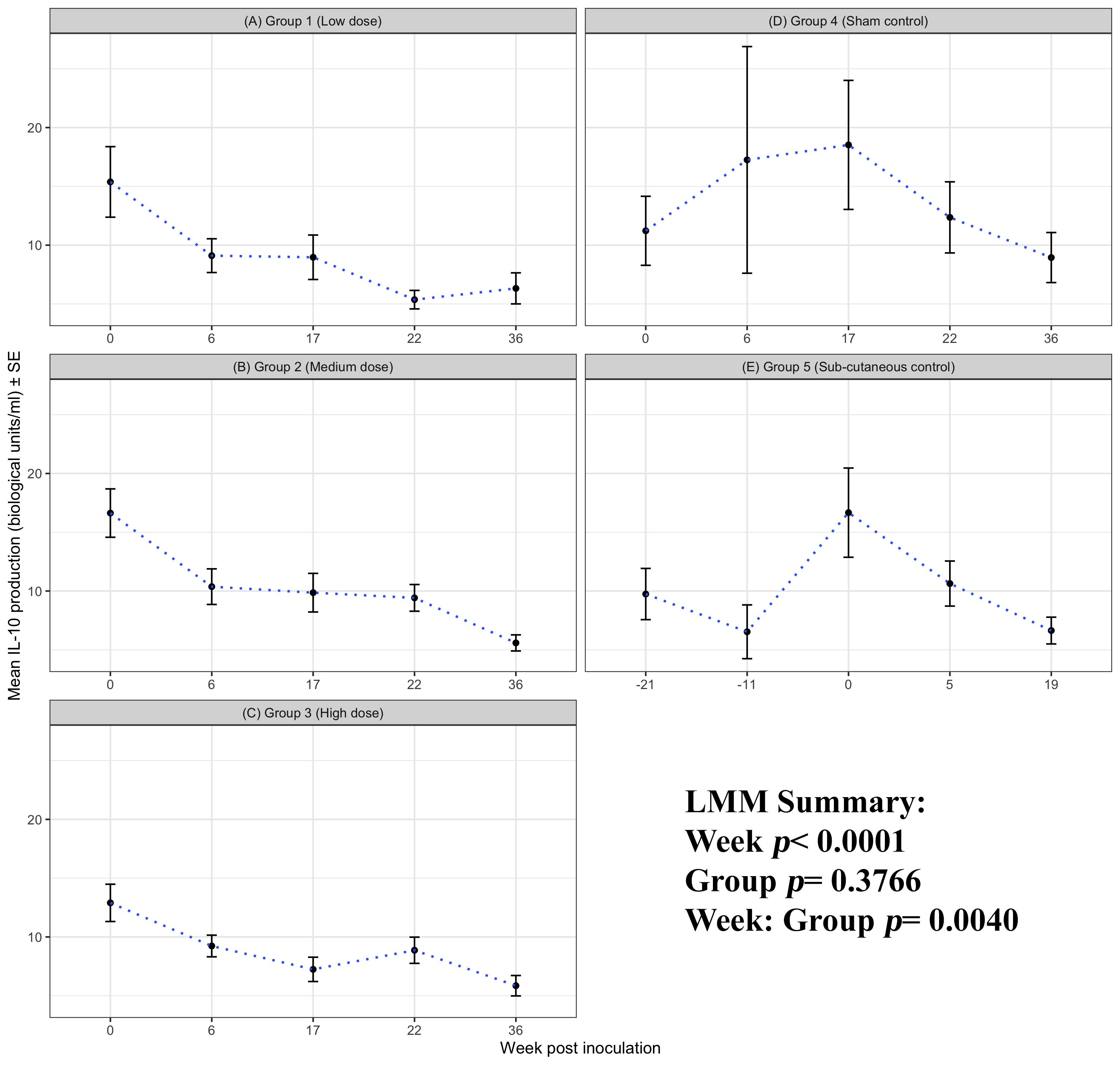

Supplement: Supplementary file 4 — Additional file 4. IL-10 production to ConA. Peripheral blood mononuclear cells from the 67 sheep in the five experimental groups were purified from whole blood (as described in section “Blood collection and peripheral blood mononuclear cell (PBMC) preparation”) on five occasions and set up in lymphocyte stimulation assays in vitro with the mitogen Concanavalin A (ConA) (set up as described in section “Lymphocyte stimulation assays”). One set of the duplicate plates were harvested for culture supernatants after 96 h and analysed for interleukin (IL)-10 production (as described in section “Cytokine ELISAs”). The datasets from each experimental group are presented in individual line graphs (A–E). The data points are the arithmetic mean values for each cellular bleed and the error bars represent the standard error of the mean (SEM). The x axis represents the weeks post inoculation with Chlamydia abortus (intranasal inoculation (i/n) Groups 1–3 with i/n sham control Group 4; and sub-cutaneous inoculation (s/c) Group 5). The week numbering for groups 1–4 are consistent in relation to i/n whereas group 5 is in relation to s/c. The y axis represents the arithmetic mean IL-10 production in biological units/millilitre amounts. (A) Group 1 (low dose), (B) Group 2 (medium dose), (C) Group 3 (high dose), (D) Group 4 (sham control) and (E) Group 5 (sub-cutaneous control). The statistics summarised in the figure been derived from Linear Mixed Modelling (LMM) as described in detail in section “Statistical analyses”. [file 13567_2020_798_MOESM4_ESM.tif]

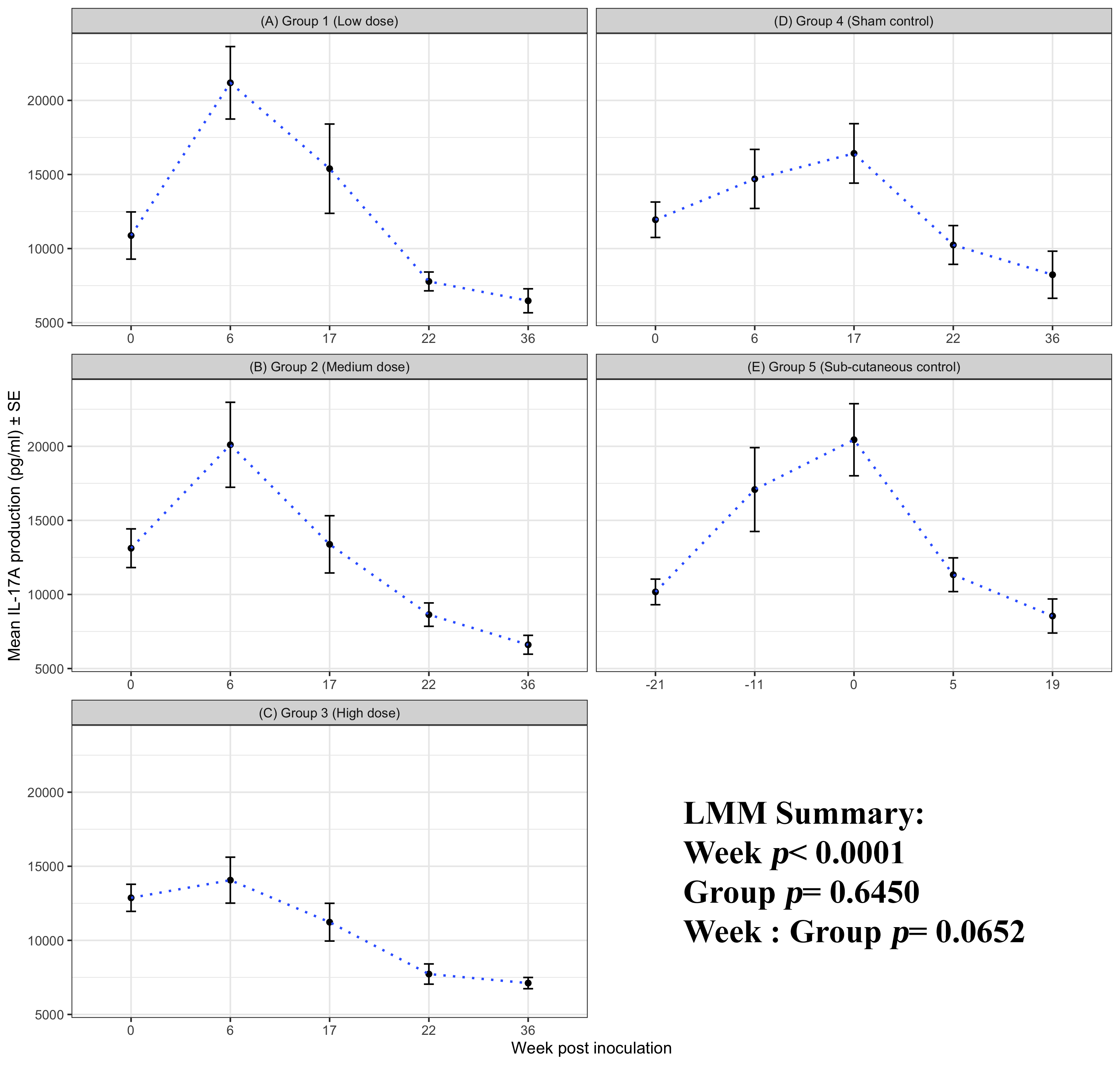

Supplement: Supplementary file 5 — Additional file 5. IL-17A production to ConA. Peripheral blood mononuclear cells from the 67 sheep in the five experimental groups were purified from whole blood (as described in section “Blood collection and peripheral blood mononuclear cell (PBMC) preparation”) on five occasions and set up in lymphocyte stimulation assays in vitro with the mitogen Concanavalin A (ConA) (set up as described in section “Lymphocyte stimulation assays”). One set of the duplicate plates were harvested for culture supernatants after 96 h and analysed for interleukin (IL)-17A production (as described in section “Cytokine ELISAs”). The datasets from each experimental group are presented in individual line graphs (A–E). The data points are the arithmetic mean values for each cellular bleed and the error bars represent the standard error of the mean (SEM). The x axis represents the weeks post inoculation with Chlamydia abortus (intranasal inoculation (i/n) Groups 1–3 with i/n sham control Group 4; and sub-cutaneous inoculation (s/c) Group 5). The week numbering for groups 1–4 are consistent in relation to i/n whereas group 5 is in relation to s/c. The y axis represents the arithmetic mean IL-17A production in picogram/millilitre concentrations. (A) Group 1 (low dose), (B) Group 2 (medium dose), (C) Group 3 (high dose), (D) Group 4 (sham control) and (E) Group 5 (sub-cutaneous control). The statistics summarised in the figure been derived from Linear Mixed Modelling (LMM) as described in detail in section “Statistical analyses”. [file 13567_2020_798_MOESM5_ESM.tif]
