# Supplementary material for: Defining immune correlates during latent and active chlamydial infection in sheep
Source: Vet Res. 2020 Jun 1;51:75. doi: 10.1186/s13567-020-00798-6 (PMC7268686; doi:10.1186/s13567-020-00798-6)
Supplement: Supplementary file 6 — Additional file 6. Summary of estimates from linear mixed modelling of the cellularChlamydia abortusantigen-driven cellular proliferation, interferon-gamma production, interleukin (IL)-10 and IL-17A production in relation to pregnancy outcome. Here are the analysis of variance tables from the linear mixed model (LMM) fits relating to each immunological parameter graphically represented in the section “Analysis of cellular immune correlates with outcome of pregnancy”. The tables refer to (A) estimates of the statistical significance of the fixed-effects terms of the models, including week, treatment group, pregnancy outcome (term called Foetus: either lambed or aborted) and its potential 2-level and 3-level interactions; and (B) post hoc comparisons between groups for each pregnancy outcome from the model fits. Note that the comparisons are made between groups 1 (low dose), group 2 (medium dose), and the challenge control group 5 (s/c control) only as explained in the section “Statistical analyses”. [file 13567_2020_798_MOESM6_ESM.docx]

**Cellular Proliferation:**

numDF denDF F-value *p*-value

(Intercept) 1 136 0.00000 1.0000

Week 4 136 36.61012 <.0001

Group 2 34 1.12486 0.3365

Foetus 1 34 3.52166 0.0692

Week:Group 8 136 2.52320 0.0137

Week:Foetus 4 136 1.44930 0.2212

Group:Foetus 2 34 0.67427 0.5162

Week:Group:Foetus 8 136 0.42487 0.9045

Comparisons between treatment groups for each abortion outcome

Foetus = Aborted:

contrast estimate SE df t.ratio *p*.value

1 - 2 -0.2975 0.250 34 -1.189 0.3641

1 - 5 0.2911 0.385 34 0.757 0.4544

2 - 5 0.5886 0.377 34 1.560 0.3641

Foetus = Lambed:

contrast estimate SE df t.ratio *p*.value

1 - 2 -0.3056 0.337 34 -0.906 0.6695

1 - 5 -0.3184 0.413 34 -0.770 0.6695

2 - 5 -0.0127 0.413 34 -0.031 0.9756

Results are averaged over the levels of: Week

Degrees-of-freedom method: containment

*P* value adjustment: fdr method for 3 tests

**IFN-γ production:**

(Intercept) 1 136 1.362934 0.2451

Week 4 136 27.755809 <.0001

Group 2 34 1.785178 0.1831

Foetus 1 34 0.001387 0.9705

Week:Group 8 136 5.230932 <.0001

Week:Foetus 4 136 0.708886 0.5872

Group:Foetus 2 34 0.119655 0.8876

Week:Group:Foetus 8 136 1.304907 0.2461

Comparisons between treatment groups for each abortion outcome

Foetus = Aborted:

contrast estimate SE df t.ratio *p*.value

1 - 2 -0.4671 0.271 34 -1.725 0.2810

1 - 5 -0.1906 0.416 34 -0.458 0.6500

2 - 5 0.2765 0.408 34 0.677 0.6500

Foetus = Lambed:

contrast estimate SE df t.ratio *p*.value

1 - 2 -0.3032 0.365 34 -0.830 0.7762

1 - 5 -0.2926 0.447 34 -0.654 0.7762

2 - 5 0.0106 0.447 34 0.024 0.9812

Results are averaged over the levels of: Week

Degrees-of-freedom method: containment

*P* value adjustment: fdr method for 3 tests

**IL-10 production:**

numDF denDF F-value p-value

(Intercept) 1 136 2.633895 0.1069

Week 4 136 15.605115 <.0001

Group 2 34 1.281398 0.2907

Foetus 1 34 0.835537 0.3671

Week:Group 8 136 2.638464 0.0102

Week:Foetus 4 136 0.773084 0.5445

Group:Foetus 2 34 0.084757 0.9189

Week:Group:Foetus 8 136 1.306987 0.2451

Comparisons between treatment groups for each abortion outcome

Foetus = Aborted:

contrast estimate SE df t.ratio *p*.value

1 - 2 -0.2726 0.258 34 -1.057 0.8222

1 - 5 -0.0368 0.396 34 -0.093 0.9267

2 - 5 0.2358 0.389 34 0.607 0.8222

Foetus = Lambed:

contrast estimate SE df t.ratio *p*.value

1 - 2 -0.4038 0.348 34 -1.161 0.7273

1 - 5 -0.2541 0.426 34 -0.597 0.7273

2 - 5 0.1497 0.426 34 0.352 0.7273

Results are averaged over the levels of: Week

Degrees-of-freedom method: containment

*P* value adjustment: fdr method for 3 tests

**IL-17A production:**

numDF denDF F-value p-value

(Intercept) 1 136 0.035883 0.8500

Week 4 136 3.341779 0.0121

Group 2 34 0.949449 0.3970

Foetus 1 34 0.581539 0.4510

Week:Group 8 136 1.944279 0.0582

Week:Foetus 4 136 1.257102 0.2900

Group:Foetus 2 34 1.192471 0.3158

Week:Group:Foetus 8 136 0.186269 0.9925

Comparisons between treatment groups for each abortion outcome

Foetus = Aborted:

contrast estimate SE df t.ratio *p*.value

1 - 2 -0.325 0.351 34 -0.926 0.5414

1 - 5 -0.581 0.539 34 -1.077 0.5414

2 - 5 -0.256 0.528 34 -0.484 0.6312

Foetus = Lambed:

contrast estimate SE df t.ratio *p*.value

1 - 2 0.577 0.473 34 1.221 0.3455

1 - 5 -0.253 0.579 34 -0.437 0.6647

2 - 5 -0.830 0.579 34 -1.434 0.3455

Results are averaged over the levels of: Week

Degrees-of-freedom method: containment

*P* value adjustment: fdr method for 3 tests
